# Copper-Bearing Metal-Organic Framework with Mucus-Penetrating Function for the Multi-Effective Clearance of Mucosal Colonized *Helicobacter pylori*

**DOI:** 10.34133/research.0358

**Published:** 2024-05-22

**Authors:** Chunxi Shu, Wei Zhang, Yiwei Zhang, Yu Li, Xinbo Xu, Yanan Zhou, Yue Zhang, Qin Zhong, Cong He, Yin Zhu, Xiaolei Wang

**Affiliations:** ^1^ Department of Gastroenterology, The First Affiliated Hospital, Jiangxi Medical College Nanchang University, Nanchang 330006, China.; ^2^ Postdoctoral Innovation Practice Base, The First Affiliated Hospital, Jiangxi Medical College, Nanchang University, Nanchang 330006, China.; ^3^ The National Engineering Research Center for Bioengineering Drugs and the Technologies, Institute of Translational Medicine, Nanchang University, Nanchang 330088, China.

## Abstract

*Helicobacter pylori* colonizes over 50% of people worldwide. Biofilm formation through penetrating gastric mucus and resistance acquired by *H. pylori* markedly reduces the efficacy of traditional antibiotics. The present triple therapy and bismuth-based quadruple therapy inevitably causes intestinal flora disturbance and fails to address the excessive *H. pylori*-triggered inflammatory response. Herein, a mucus-permeable therapeutic platform (Cu-MOF@NF) that consists of copper-bearing metal-organic framework (Cu-MOF) loaded with nitrogen-doped carbon dots and naturally active polysaccharide fucoidan is developed. The experimental results demonstrate that Cu-MOF@NF can penetrate the mucus layer and hinder *H. pylori* from adhering on gastric epithelial cells of the stomach. Notably, released Cu^2+^ can degrade the polysaccharides in the biofilm and interfere with the cyclic growing mode of “bacterioplankton ↔ biofilm”, thereby preventing recurrent and persistent infection. Compared with traditional triple therapy, the Cu-MOF@NF not only possesses impressive antibacterial effect (even include multidrug-resistant strains), but also improves the inflammatory microenvironment without disrupting the balance of intestinal flora, providing a more efficient, safe, and antibiotic-free new approach to eradicating *H. pylori*.

## Introduction

**Fig. 1.**
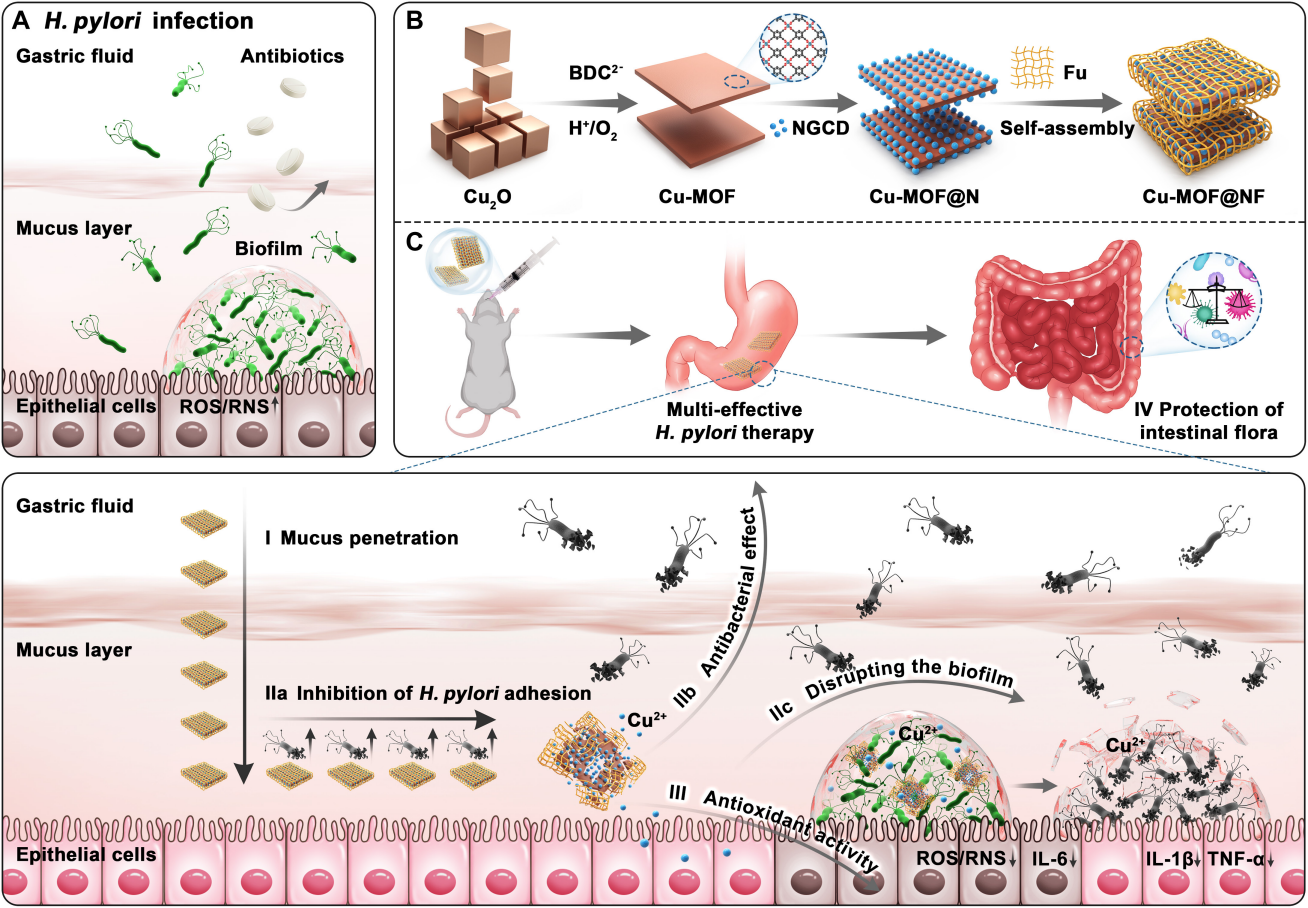
Schematic illustration of the fabrication process and curative action of Cu-MOF@NF in *H. pylori* infection. (A) The process of *H. pylori* infection of the gastric mucosa. (B) The fabrication process of Cu-MOF@NF. (C) The capabilities of Cu-MOF@NF, including penetrating the gastric mucus layer (I), eliminating *H. pylori* planktonic bacteria and biofilms (II), regulating the inflammatory microenvironment (III), and maintaining intestinal flora homeostasis (IV).

*Helicobacter pylori*, a gram-negative oncogenic pathogen, has infected about 50% of people worldwide. Its colonization of the gastric mucosa can induce chronic gastritis and potentially progress to chronic atrophic gastritis, intestinal metaplasia, and heterotrophic hyperplasia, and ultimately develop into gastric cancer [1–3]. Studies have suggested that approximately 90% of non-cardia cancers are associated with *H. pylori* infection [4]. At present, traditional triple therapy and bismuth-based quadruple therapy are used clinically to manage *H. pylori* infection [5]. All of these approaches suffer from the inability of the drugs to penetrate the gastric mucus layer effectively, clear the biofilm [6,7], and alleviate the inflammatory response induced by the infection (Fig. 1A) [8,9]. Moreover, antibiotic-based therapies tend to disrupt the homeostasis of intestinal flora, leading to the emergence of various intestinal diseases [10]. Therefore, a novel antibiotic alternative therapy is urgently needed to attain the following 4 requirements: (a) Penetrating the gastric mucus layer effectively. Both planktonic *H. pylori* and formed biofilm are colonized beneath the mucus layer, and the drug is required to break through the mucus obstacle prior to reaching infected sites [11]. (b) Blocking the cyclic growing mode of “bacterioplankton ↔ biofilm” to prevent persistent infection. Most *H. pylori* colonize beneath the gastric mucus and form a cyclic growing mode of “bacterioplankton ↔ biofilm” [12], which probably explains an element of persistent *H. pylori* infection. As the planktonic state, *H. pylori* is an individual free-floating bacterium that can move and colonize new areas of the stomach. Once the aggregates are formed, they will transform into the biofilm state [13]. Generally, the formed biofilm makes *H. pylori* significantly less susceptible to antibiotics and more aggressive to the immune system. Therefore, failure to simultaneously eradicate *H. pylori* in both planktonic and biofilm states can result in persistent infection [12,14]. Given the aforementioned reasons, new therapies are expected to possess the following 2 functions compared with the traditional antibiotic treatment: (c) scavenging reactive oxygen species (ROS) and regulating inflammatory response, and (d) preserving intestinal flora.

To address the above problems, Cu-MOF@NF, an *H. pylori* treatment platform with mucus penetration function, was designed (Fig. 1B). It consisted of a copper-bearing metal-organic framework (Cu-MOF) loaded by nitrogen-doped carbon dots (NGCDs) and naturally active polysaccharide fucoidan (FU), offering the following 4 major advantages: (a) Penetrating the gastric mucus layer effectively. It is worth mentioning that the FU coating has good biocompatibility and a negative charge, allowing the drug to adhere and penetrate the mucus layer more effectively [15,16]. NGCD nanoparticles adsorbed in Cu-MOF pores and the surface coating FU functionalized by non-covalent bonding interactions could improve the hydrophily and stability of Cu-MOF@N, which promoted the mucus-permeable function of Cu-MOF@NF. (b) Blocking the periodic growth pattern of “planktonic bacteria ↔ biofilm” to prevent persistent infection. After penetrating the mucus layer, FU on the surface blocks *H. pylori* blood group antigen-binding adhesin (BabA) from binding to the Lewis^b^ antigens of the host cell and competitively inhibits the adhesion of bacteria, thereby inhibiting biofilm regeneration [17]. After reaching the site of *H. pylori* colonization beneath the mucus layer, the Cu^2+^ released by the degradation of Cu-MOF@NF could penetrate the biofilm and degrade the polysaccharides in the biofilm matrix. Meanwhile, it could efficiently kill *H. pylori* in the biofilm, as well as planktonic *H. pylori*, by reducing the adenosine triphosphate (ATP) level in the bacteria and promoting bacterial membrane permeability, thereby blocking the growth cycle of “planktonic bacteria ↔ biofilm” and preventing recurrent and persistent infection. (c) Scavenging ROS and regulating inflammatory response. Recent studies have shown that carbon dots can effectively eliminate intracellular ROS and have excellent antioxidant activity [18–20]. Nitrogen, which has the advantages of small atomic weight, safety, economy, environmental friendliness, and low toxicity, is chosen as the heteroatom. Doping nitrogen heteroatoms can alter the atomic structure of carbon dots, which improves the antioxidant performance and reduces the toxicity of carbon dots [21,22]. Moreover, carbon dots can down-regulate the expression of pro-inflammatory cytokines, thereby exerting good anti-inflammatory effects [23]. NGCD released by Cu-MOF@NF improved the inflammatory microenvironment by acting on *H. pylori*-infected gastric epithelial cells, scavenging excessive oxygen free radicals to attenuate oxidative stress-induced gastric mucosal epithelial cell damage, along with inhibiting the expression of pro-inflammatory cytokines like interleukin-1β (IL-1β), IL-6, and tumor necrosis factor-α (TNF-α). (d) Preserving intestinal flora. The system we constructed had 3 main properties: efficient penetration of gastric mucus, multi-effective clearance of *H. pylori*, and no need for antibiotics; thus, the platform could also theoretically avoid antibiotic-induced imbalances in the intestinal flora (Fig. 1C). Subsequently, an array of in vivo and in vitro experiments were designed to validate the effectiveness of the system.

## Results and Discussion

### Synthesis and characterization of Cu-MOF@NF

**Fig. 2.**
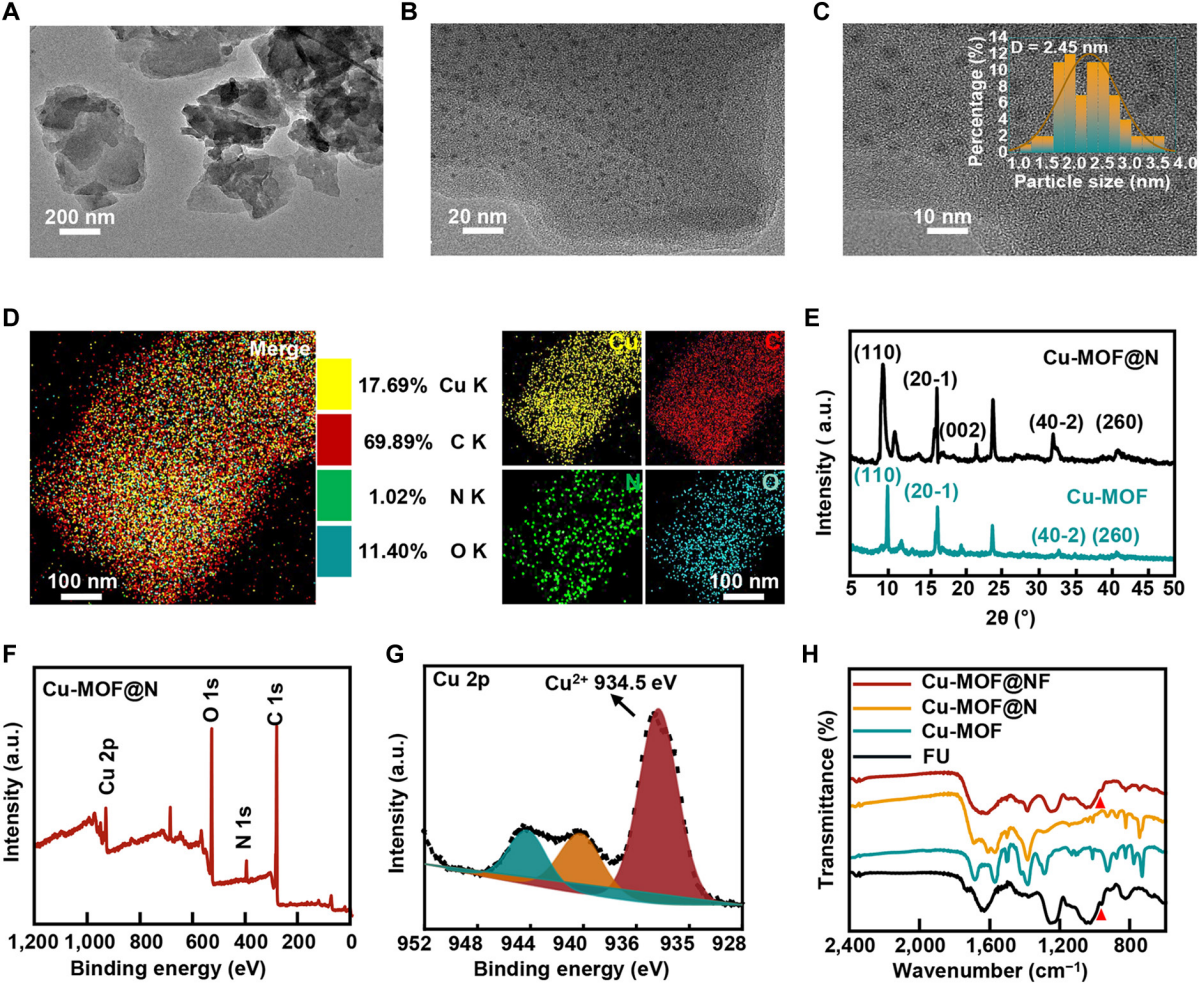
Characterization of Cu-MOF@NF. (A) Low-magnification TEM image of Cu-MOF@N. (B) High-magnification TEM image of Cu-MOF@N, where the illustration showed the size distributions of the corresponding NGCD. (C) The corresponding sizes and distributions of the NGCD. (D) Corresponding elemental mapping of Cu-MOF@N. (E) XRD patterns of Cu-MOF and Cu-MOF@N. (F) Low-resolution XPS spectrum of Cu-MOF@N. (G) High-resolution XPS spectrum of Cu 2p of Cu-MOF@N. (H) The FTIR spectra of Cu-MOF@NF, Cu-MOF@N, Cu-MOF, and FU.

Copper compounds have been extensively used for treating skin diseases, syphilis, and tuberculosis in the pre-antibiotic era, and its antimicrobial capabilities are also well recognized in modern healthcare [24,25]. Unlike gold, silver, and platinum, copper is an essential trace element for the human body, and excess copper is relatively easy to eliminate [26]. Firstly, scanning electron microscopy (SEM) and atomic force microscopy (AFM) images revealed that the Cu-MOF was in the form of square flakes, with the average margin length of Cu-MOF being 300 to 500 nm (Figs. S1 and S2). The x-ray diffraction (XRD) pattern of the NGCD clearly displayed a broad diffraction peak at 2θ = 23.12°, in accordance with earlier studies (Fig. S3) [27], indicating the successful synthesis of NGCD. Subsequently, coordinatively unsaturated metal centers in Cu-MOF potentially formed coordination bonds with the carbonyl group of the alkenone group in the NGCD, enabling the chemisorption of Cu-MOF with NGCD nanoparticles [28–30]. The transmission electron microscopy (TEM) demonstrated that the Cu-MOF nanosheets could preserve their morphology after adsorbing NGCD, and the NGCD of 2.45 nm average size were well spread throughout the outer surface of the Cu-MOF nanosheets (Fig. 2A to C). The TEM elemental mapping was used to visualize the distribution of Cu, C, N, and O elements (Fig. 2D). The crystal structure of Cu-MOF@N was determined by XRD, where the crystallographic peaks of Cu-MOF@N coincided with the (1 1 0), (2 0 −1), and (4 0 −2) major crystallographic planes of Cu-MOF alone and the (0 0 2) crystal peaks of NGCD (Fig. 2E). Secondly, the valence states of Cu elements in Cu-MOF@N were investigated by x-ray photoelectron spectroscopy (XPS). As shown in Fig. 2F and G, for the Cu-MOF nanosheet sample, the characteristic XPS peak of Cu 2p_3/2_ was detected at the binding energy of 934.5 eV. In Fig. S4A, the C 1s spectrum exhibited 2 characteristic peaks at 288.5 eV and 284.8 eV, corresponding to sp2 hybridized carbon (N−C=N) and graphene carbon (C=C) [31], respectively. Furthermore, the characteristic peak at 398.05 eV matched with the N 1S binding energy of nitrogen bonds (C−N and N−H) in the amide or amino group of NGCD (Fig. S4B) [21], which also indicated that NGCDs were successfully adsorbed on Cu-MOF nanosheets. After that, FU was modified on the surface of the particles by hydrophobic interactions (van der Waals forces). As a surface coating, FU could effectively improve the water solubility, stability, and mucus penetration function of Cu-MOF@N. The Fourier transform infrared (FTIR) spectra of both FU and Cu-MOF@NF showed sugar ring stretching vibration peaks at around 968 cm^−1^, which was in accordance with the vibration of C−O−C bonds in the α-glucan chains of FU polysaccharides [32], indicating the successful wrapping of FU (Fig. 2H and Fig. S5). Considering the special gastric environment and the gastric emptying time, we co-incubated Cu-MOF, Cu-MOF@N, and Cu-MOF@NF with simulated gastric fluid (SGF) for 4 h. Figure S6 showed that Cu-MOF and Cu-MOF@N were unstable in the SGF, accompanied by the deposition of materials. In contrast, Cu-MOF@NF was almost unchanged in the SGF, and the solution was homogeneous. It was assumed that this resulted from the lack of protective layers of Cu-MOF and Cu-MOF@N, which induced the degradation of the organic framework of Cu-MOF under acidic conditions, resulting in a certain degree of agglomeration. The Cu content in Cu-MOF@NF was quantified to be about 5.5% using inductively coupled plasma-atomic emission spectrometry (ICP-AES) (Table S1).

### The ability of Cu-MOF@NF in mucus penetration and *H. pylori* adhesion inhibition

**Fig. 3.**
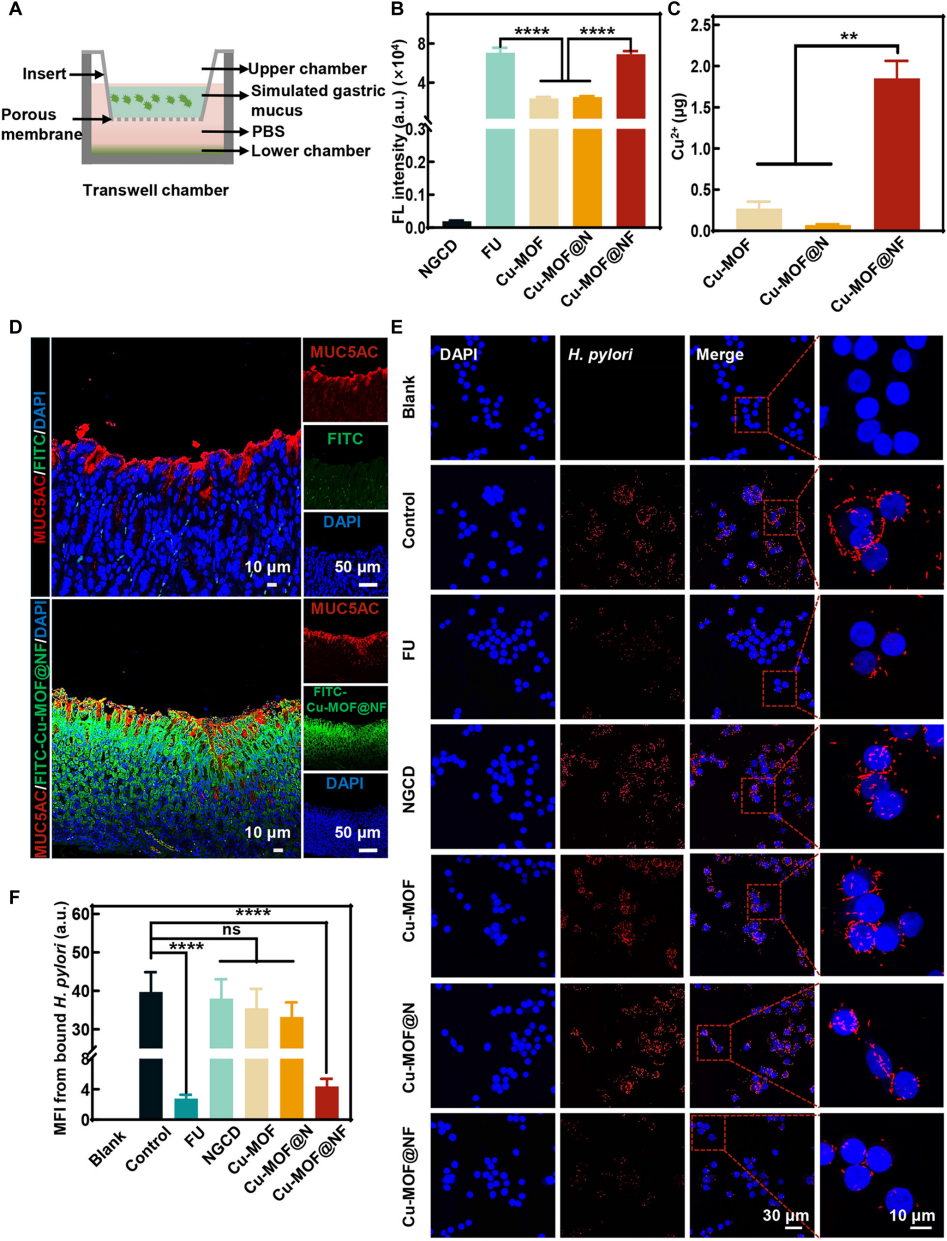
In vitro nanoparticles’ mucus penetration and inhibition of *H. pylori* adhesion. (A) Schematic diagram of the penetration of Cu-MOF@NF nanoparticles through the gastric mucus layer in transwell. (B) Fluorescence quantification of each group of nanoparticles from the lower chamber of the transwell in simulated gastric mucus. (C) Quantification of the Cu^2+^ of the nanoparticles after penetration of the agar block. (D) CLSM images of the mucus penetration of Cu-MOF@NF in the stomachs of mice. (E) CLSM images of *H. pylori* bound with HFE145 cells after incubation with different nanoparticles with 25 μg ml^−1^ concentration. (F) Quantitative analysis of the mean fluorescence intensity (MFI) of *H. pylori* in (E). Data are means ± SD (*n* ≥ 3). ns indicates not significant, with *P* ≥ 0.05. **P < 0.01, ****P < 0.0001.

Nanomaterials can only exert their effects after penetrating the mucus layer and reaching the colonization site of *H. pylori*. Generally, compounds with weak negative charges or near-neutral charges and hydrophilicity interact minimally with mucus and thus readily permeate the mucus layer [33–37]. When FU wrapped Cu-MOF@N nanoparticles through hydrophobic interactions (van der Waals forces), the Zeta potential value of nanoparticles in SGF became the desired weak negative charge and showed no significant difference compared to the Zeta potential value in phosphate-buffered saline (PBS) (Fig. S7), which reduced the interaction of Cu-MOF@NF with the mucus layer, allowing for penetrating the mucus layer more easily. We further utilized an ICP-AES instrument to measure the release curves of Cu^2+^ at different pH values. The results indicated that Cu^2+^ was released under different pH conditions (Fig. S8). Therefore, the FU coating designed here not only improved the stability of Cu-MOF@NF, but also increased its permeability and ability to inhibit bacterial adhesion. Furthermore, the transwell device was used to study the mucus permeability of Cu-MOF@NF, and the results were quantified by measuring the fluorescence intensity of fluorescein isothiocyanate (FITC)-labeled material permeating simulated gastric mucus in the lower chamber (Fig. 3A). As presented in Fig. 3B, stronger fluorescence and significant differences were detected in FU and Cu-MOF@NF groups relative to other groups. Similarly, the SGF was placed on the hardened agarose gel, and then the samples were stained with Coomassie brilliant blue and deposited on the surface of the SGF. Over time, it could be observed that FU and Cu-MOF@NF penetrated the agarose gel more rapidly than the other groups (Fig. S9). The amount of Cu^2+^ in the bottom layer of the agarose gel was subsequently analyzed by ICP-AES to quantify the mucus permeability. We observed that the Cu-MOF@NF group had the highest level of Cu^2+^ (Fig. 3C), which indicated that the FU-coated nanoparticles had better mucus permeability than the bare nanoparticles. Afterwards, to investigate the penetration of Cu-MOF@NF in vivo, the stomachs of mice were taken for immunofluorescence examination after 2 h of gavage with FITC-labeled Cu-MOF@NF (FITC-Cu-MOF@NF), and mucus layers of the stomachs were co-localized with MUC5AC mucosal glycoprotein-specific antibodies [38]. Under confocal laser scanning microscopy (CLSM), it could be recognized that the Cu-MOF@NF group had a large amount of FITC-Cu-MOF@NF in the lower gastric mucus layer where the MUC5AC antibody was expressed in comparison to the FITC gavage (control) group (Fig. 3D). This suggested the good ability of Cu-MOF@NF to penetrate the gastric mucus of mice, which provided good feasibility for in vivo treatment in mice. Then, the antibacterial properties of Cu-MOF@NF were firstly assessed in vitro. From Fig. S10, the antibacterial effects of Cu-MOF@NF against *H. pylori* strain ATCC 43504 and rodent-adapted CagA^+^ *H. pylori* strain pre-murine Sydney Strain 1 (PMSS1) were concentration-dependent. There was no statistical difference in antimicrobial effect between 180 μg ml^−1^ and 200 μg ml^−1^ concentrations, and thus the optimal bacteriostatic concentration was 180 μg ml^−1^. To validate the suppressive role of Cu-MOF@NF against adhesion of *H. pylori*, it was investigated by locating *H. pylori* with its antibodies through immunofluorescence assay, which studied the adhesion of *H. pylori* on the gastric mucosa cell line (HFE145) and human gastric epithelium (GES-1) cells after treating with 25 μg ml^−1^ Cu-MOF@NF. It has been demonstrated that Cu-MOF@NF has almost no antimicrobial effect at a density of 25 μg ml^−1^, which therefore excludes the total number of bacteria becoming less due to the antimicrobial effect of the material itself (Fig. S11). It was observed that there was good cell growth and no bacterial adhesion in the blank group, whereas there was aggregation of *H. pylori* (red) surrounding the cells (blue) in the control group, implying that the bacteria were widely adhering to the cell surface. Notably, *H. pylori* adhesion was hardly observed in the FU and Cu-MOF@NF groups (Fig. 3E and Fig. S12A). Further quantification of fluorescence intensity showed a significant reduction in *H. pylori* adhesion when treated with FU and Cu-MOF@NF (Fig. 3F and Fig. S12B). Namely, as the sulfate polysaccharide containing the rock-algae glycosyl group, FU prevents cell adhesion of *H. pylori* by decreasing its interaction with epithelial cells. The probable mechanism involved the competitive binding of FU to BabA of *H. pylori* [39,40].

### Antibacterial activity and mechanism of Cu-MOF@NF

**Fig. 4.**
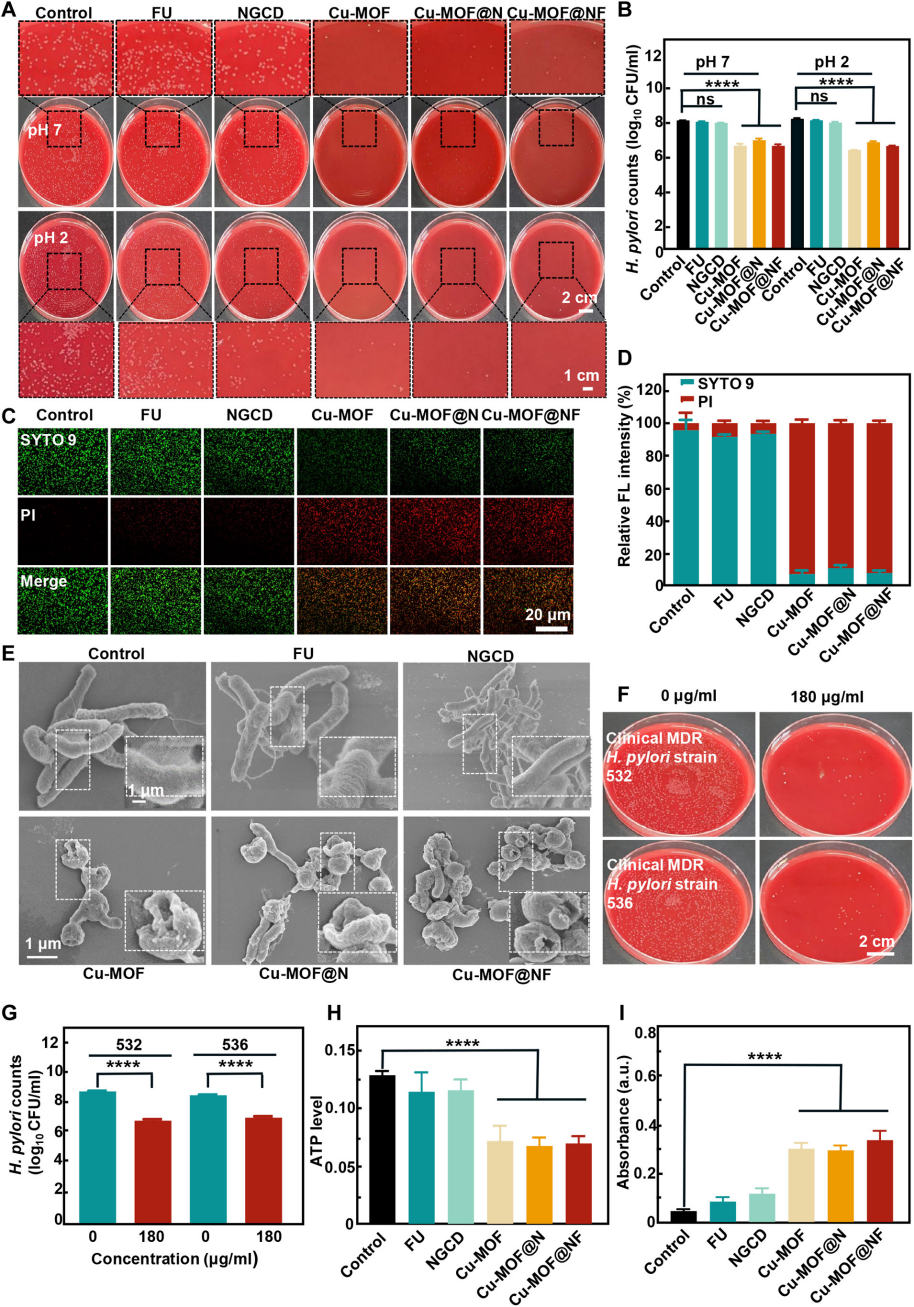
In vitro study of the antimicrobial activity and mechanism of nanoparticles. (A) Images of *H. pylori* colony plates after treatment with different samples of 180 μg ml^−1^ concentration under different pH conditions. (B) *H. pylori* growth counted in CFUs per milliliter in (A). (C) Live (SYTO 9)/dead (PI) fluorescence images of *H. pylori* exposed to each group of nanoparticles with 180 μg ml^−1^ concentration. (D) Fluorescence intensity of SYTO 9 and PI of *H. pylori* in different treatment groups at the concentration of 180 μg ml^−1^. (E) SEM plots of changes in physiology and morphology of *H. pylori* after different treatments with 180 μg ml^−1^ concentration. (F) Colony plate plots of clinical drug-resistant strains 532 and 536 after treatment with 180 μg ml^−1^Cu-MOF@NF. (G) *H. pylori* growth counted in CFUs per milliliter in (F). (H) ATP metabolism level of *H. pylori* after treatment with nanoparticles with 180 μg ml^−1^ concentration in each group. (I) Changes in the permeability of the bacterial membranes of *H. pylori* as determined by β-galactosidase. Data are means ± SD (*n* ≥ 3). ns indicates not significant, with *P* ≥ 0.05. ****P < 0.0001.

Considering that the pH of human gastric acid ranges from 1 to 2, the antibacterial effect of each group of materials under different pH conditions was investigated. By measuring the colony-forming units (CFUs) on agar plates (Fig. 4A and B), it was observed that the Cu-MOF-containing groups exhibited significant antibacterial activity at both pH 2 and pH 7, whereas the FU and NGCD groups showed almost no antibacterial effect. This manifested that the antibacterial impact of the materials was mainly attributed to Cu-MOF, although the effect of the bare Cu-MOF group was slightly more powerful in comparison with that of the Cu-MOF@N and Cu-MOF@NF groups. This difference might be due to the release of more Cu^2+^ in the bare Cu-MOF group at the same concentration. The antibacterial activity of the material was further analyzed by live/dead bacterial staining. The bacteria treated with Cu-MOF-based materials displayed a large amount of red fluorescence under fluorescence microscopy (Fig. 4C and D), further demonstrating the excellent antibacterial activity of the Cu-MOF-containing groups against *H. pylori.* Additionally, SEM images exhibited more significant depression and rupture of the bacterial membrane of *H. pylori* in the Cu-MOF-containing group than in the control group (Fig. 4E). Based on the good antibacterial performance of Cu-MOF@NF, we also evaluated the antibacterial effectiveness of Cu-MOF@NF against multidrug-resistant (MDR) *H. pylori* strains 532 and 536. From the CFUs on agar plates (Fig. 4F and G), Cu-MOF@NF showed an excellent antibacterial effect against MDR *H. pylori* strains 532 and 536. Then, the antibacterial mechanism of Cu-MOF@NF was investigated. Studies have shown that the alteration of ATP levels can affect the function of the cells, and usually the ATP level decreases when the cells are apoptotic, necrotic, or in some toxic state [41]. As shown in Fig. 4H, the Cu-MOF-containing groups could significantly reduce the level of ATP compared with other groups. The results of the corresponding bacterial cell membrane permeability experiments were consistent with the above results (Fig. 4I), as the Cu-MOF-containing groups could significantly increase the bacterial membrane permeability compared with other groups. To conclude, the results demonstrated that Cu-MOF@NF could achieve highly effective antibacterial capability against *H. pylori* by decreasing ATP levels and increasing bacterial membrane permeability.

### Anti-biofilm activity of Cu-MOF@NF toward *H. pylori*

**Fig. 5.**
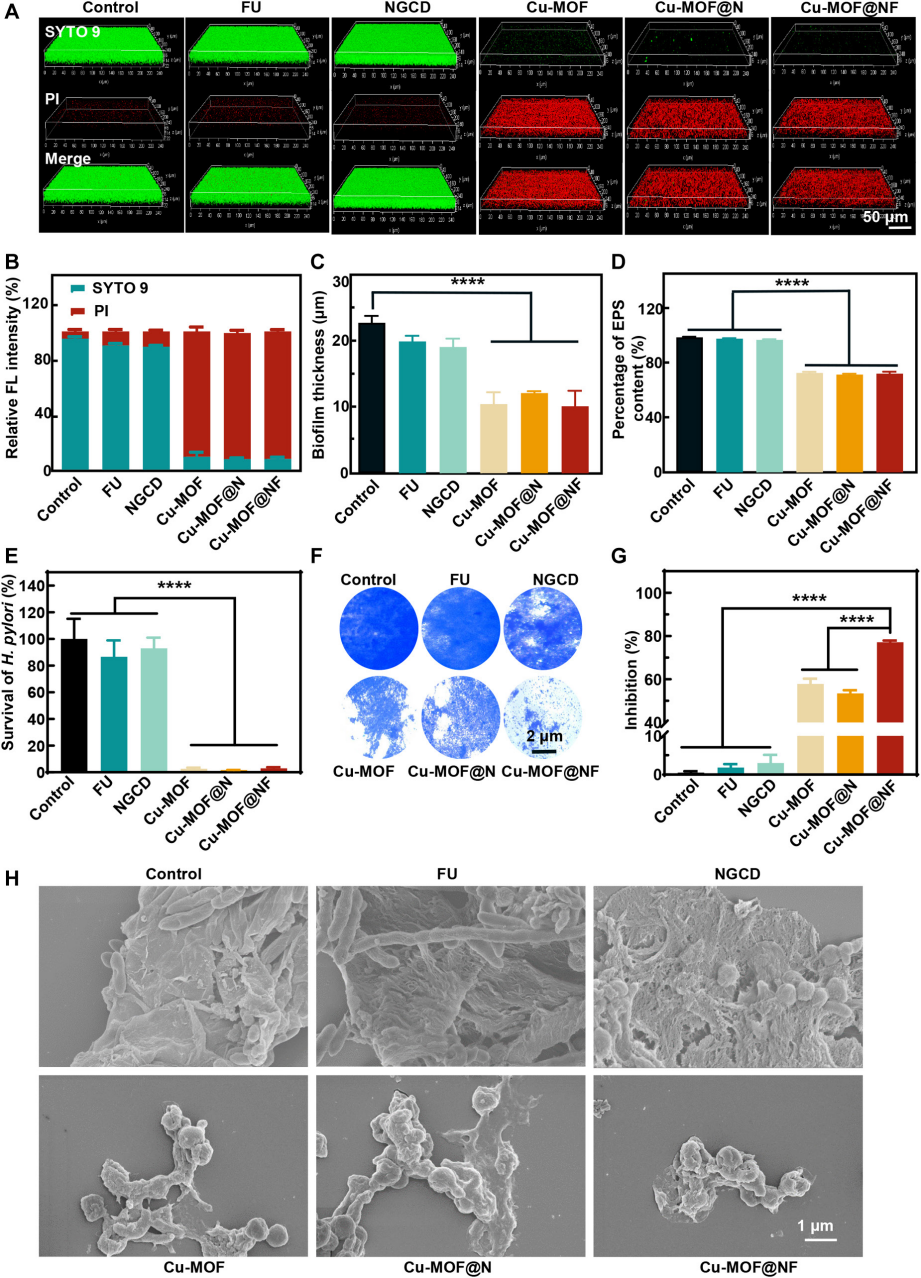
In vitro clearance of *H. pylori* biofilm by nanoparticles. (A) 3D CLSM images of *H. pylori* ATCC 43504 biofilm treated by each group of nanoparticles at the concentration of 180 μg ml^−1^. (B) Fluorescence analysis of SYTO 9 and PI in (A). (C) Thickness of mature *H. pylori* biofilm stained by SYTO 9 in 3-dimensional confocal images. (D) Concentration of polysaccharides in the EPS of the mature biofilm of *H. pylori* ATCC 43504 was determined via the phenol–sulfuric acid method after different treatments. (E) Killing properties of nanoparticles against dispersed *H. pylori* in biofilm. (F and G) Pictures and inhibition rate of crystal violet-stained mature biofilm EPS under different inhibition strategies. (H) SEM observation of *H. pylori* biofilm structure after nanoparticles treatment with 180 μg ml^−1^ concentration. Data are means ± SD (*n* ≥ 3). ****P < 0.0001.

The biofilm acts as a shelter for internal bacteria and evades the host immune system [12,42]. Therefore, if the prepared nanoparticles are able to penetrate the biofilm effectively, their killing efficiency within the biofilm can be greatly improved [10]. Based on the good antibacterial effect of Cu-MOF@NF against planktonic *H. pylori*, the disruption of biofilm by Cu-MOF@NF was evaluated next. Bacteria within the biofilm were stained for CLSM observation using green fluorescent nucleic acid stain (SYTO 9) and red fluorescent dye pyridine iodide (PI). It was observed in Fig. 5A that the biofilms of the control group, FU group, and NGCD groups showed only live *H. pylori* with uniform green fluorescence, whereas the number of dead bacteria increased significantly after treatment in the Cu-MOF-containing groups. Quantitative results on live bacterial percentage and biofilm thickness showed that the Cu-MOF-containing groups reduced the *H. pylori* in the biofilm by about 90%, and the thickness of the biofilm was markedly reduced (Fig. 5B and C), respectively. Biofilms are 3-dimensional multicellular clusters of bacteria embedded with self-secreted extracellular polymeric substances (EPS), consisting largely of polysaccharides and proteomes [43–46]. Therefore, the degradation of polysaccharides in biofilms after co-incubation with materials was investigated. As shown in Fig. 5D, the remaining polysaccharides in the biofilm were quantified by the phenol–sulfuric acid method, where the amounts of polysaccharides in the Cu-MOF-containing groups were drastically less than those in the control, FU, and NGCD groups. The significant reduction in biofilm thickness might be attributed to the degradation of polysaccharides in the biofilm. In addition, there is a cyclic growing mode of “bacterioplankton ↔ biofilm” in *H. pylori*, which induces persistent infection. That is, after Cu-MOF@NF disrupted the biofilm to release large amounts of plankton, the escaping survival bacteria would re-adhere and form new biofilm structures, causing and exacerbating a more widespread infection. Therefore, ideal therapy necessitates both biofilm disintegration and continual suppression of survival bacteria in biofilms. After incubation of nanoparticles with *H. pylori* biofilm for 24 h, the supernatant was collected and the survival rate of *H. pylori* was assessed through plate counting. The results revealed that all the dispersed *H. pylori* bacteria were eradicated following treatment with the Cu-MOF-containing groups (Fig. 5E), significantly reducing the risk of persistent infection. The challenge of regenerating biofilm remains in the presence of low levels of residual *H. pylori*. Therefore, we also evaluated the ability to inhibit the reformation of *H. pylori* biofilm using crystal violet staining, and the results suggested that the Cu-MOF-containing groups could indeed inhibit the biofilm reformation (Fig. 5F). Quantitative analysis showed that the anti-biofilm activity was remarkably enhanced in the Cu-MOF@NF group relative to the Cu-MOF and Cu-MOF@N groups (Fig. 5G). This might be ascribed to the fact that FU in the Cu-MOF@NF group could competitively inhibit the adhesion of *H. pylori*, which reduced bacterial interactions. Moreover, Cu^2+^ could exert substantial bactericidal efficiency, achieving the synergistic inhibition of biofilm regeneration. Then, the biofilm structure of *H. pylori* was directly recognized by SEM. In the control group, the biofilm structure was dense and the bacterial membrane was structurally intact, whereas the biofilm in the Cu-MOF-containing groups was conspicuously loose and diminished, and *H. pylori* was significantly globularized, with the bacterial membrane depressed (Fig. 5H). These findings suggested that Cu-MOF@NF might be a potential candidate for defense against bacterial biofilms.

### Antioxidant stress and improving the inflammatory microenvironment ability of Cu-MOF@NF

**Fig. 6.**
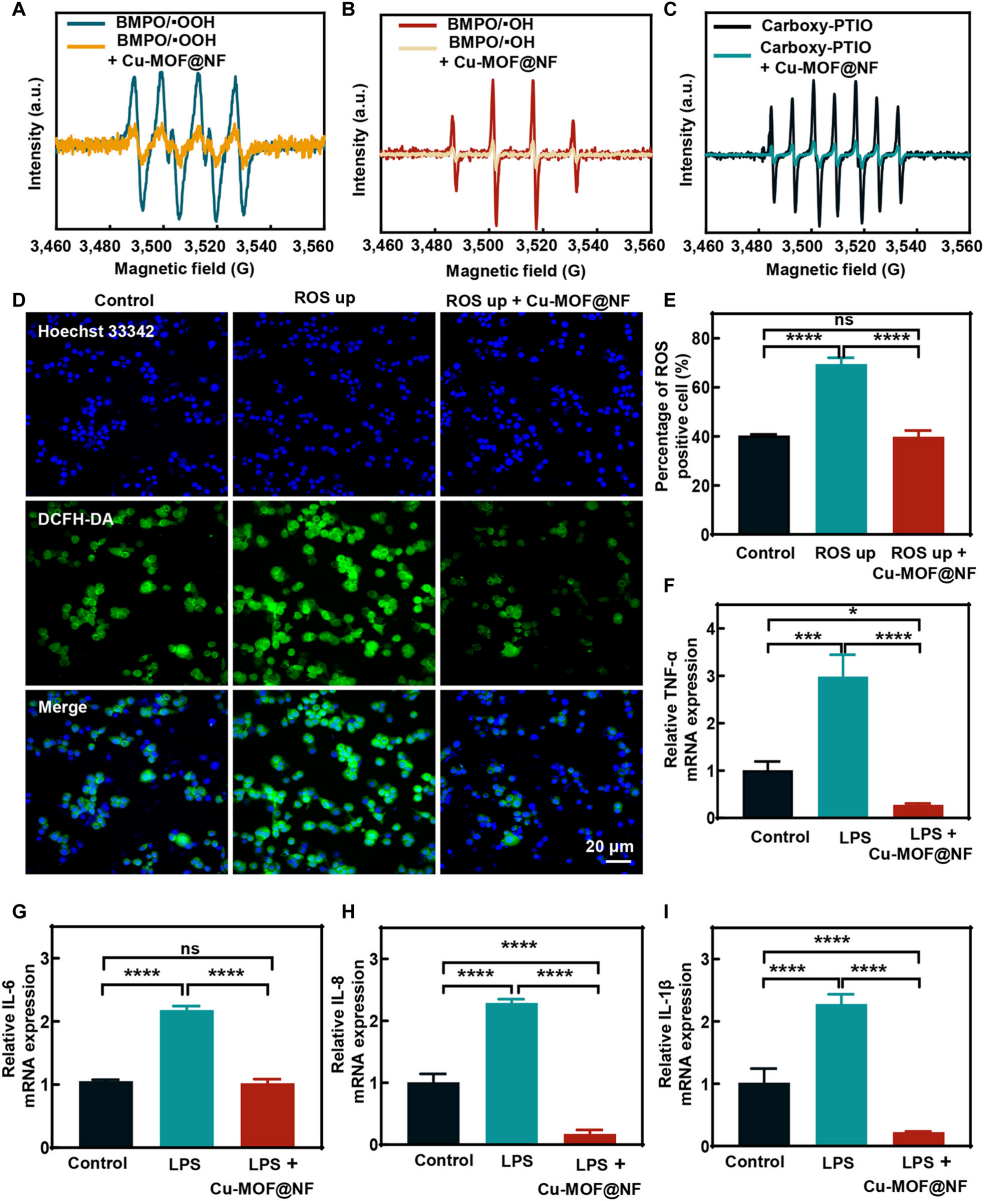
The reactive oxygen radical scavenging activity and the inflammatory microenvironment improving effect of Cu-MOF@NF. (A to C) Scavenging activities of O_2_^•**−**^, ^•^OH, and ^•^NO by Cu-MOF@NF (180 μg ml^−1^), respectively. (D) ROS levels within HFE145 cells treated by Cu-MOF@NF (180 μg ml^−1^). (E) Quantitative statistical plots of DCFH-DA in (D). (F to I) RT-qPCR assay for mRNA levels of inflammatory cytokines induced by LPS in HFE145 cells, consisting of TNF-α, IL-6, IL-8, and IL-1β. Data are means ± SD (*n* ≥ 3). ns indicates not significant, with *P* ≥ 0.05. *P < 0.05, ***P < 0.001, ****P < 0.0001.

Overexpression of inflammatory factors and oxidative stress mediated by excessive accumulation of ROS due to *H. pylori* infection is proven closely related to the development with diseases of the stomach like chronic gastritis, peptic ulcer, and even gastric cancer [47–49]. Increases in ROS and reactive nitrogen species (RNS) can lead to oxidative and antioxidant imbalances in vivo [50]. Here, the 2,2-diphenyl-1-picrylhydrazyl (DPPH) and 2,2-azinobis (3-ethylbenzthiazoline-6-sulfonate) (ABTS) methods were first performed to evaluate their free radical scavenging function. NGCD, Cu-MOF@N, and Cu-MOF@NF effectively scavenged DPPH (Fig. S13A to C). Second, the general antioxidant properties of different concentrations of NGCD, Cu-MOF@N, and Cu-MOF@NF against ROS were determined by the ABTS method (Fig. S13D and E). Moreover, the removal of ROS and RNS by Cu-MOF@NF was investigated by monitoring the individual group reactions. Electron spin resonance (ESR) was utilized to show the scavenging abilities of Cu-MOF@NF to scavenge O_2_^•**−**^, ^•^OH, and ^•^NO, respectively (Fig. 6A to C). The scavenging function of Cu-MOF@NF for ROS and RNS was due to the fact that the surface of NGCDs loaded in Cu-MOF@NF had many functional groups, such as hydroxyl, carbonyl, hydroxyl-amino groups, etc., which could directly react with free radicals, thereby transforming them into stable compounds and achieving the scavenging effect of the ROS and RNS [51,52]. Subsequently, fluorescence images of ROS up cells in the presence and absence of Cu-MOF@NF were shown on HFE145 and GES-1 using a 2,7-dichlorodihydrofluorescein diacetate (DCFH-DA) fluorescent probe. Compared with healthy cells, cells in the ROS up group showed stronger fluorescence, implying excessive ROS production. After Cu-MOF@NF treatment, the fluorescence signals were significantly weakened and close to the level of blank cells due to the effective removal of ROS (Fig. 6D and E and Fig. S14), confirming the role of Cu-MOF@NF in alleviating oxidative stress. Thereafter, the mRNA expression levels of inflammatory cytokines IL-1β, IL-6, IL-8, and TNF-α in HFE145 cells induced by lipopolysaccharide (LPS) were detected by real-time polymerase chain reaction (RT-qPCR). The findings revealed that the mRNA levels of inflammatory cytokines IL-1β, IL-6, IL-8, and TNF-α were markedly reduced after Cu-MOF@NF treatment in comparison with the LPS group, approaching the levels of blank cells (Fig. 6F to I). Thus, Cu-MOF@NF had a notable role in removing ROS and RNS as well as inhibiting inflammatory factors to alleviate inflammation. Good biocompatibility is the prerequisite for the in vivo application of biomaterials. The cytotoxicity of Cu-MOF, Cu-MOF@N, and Cu-MOF@NF was preliminarily investigated with HFE145 and GES-1 cells. Figures S15 to S17 show that the cell viability of HFE145 and GES-1 cells after 2 days of incubation with Cu-MOF, Cu-MOF@N, and Cu-MOF@NF remained above 80%, which indicated that these materials had good biosafety. Erythrocyte hemolysis test and live/dead cell staining of cells also proved its good biocompatibility (Fig. S18 and S19).

### Cu-MOF@NF treatment of *H. pylori* infection in mice

**Fig. 7.**
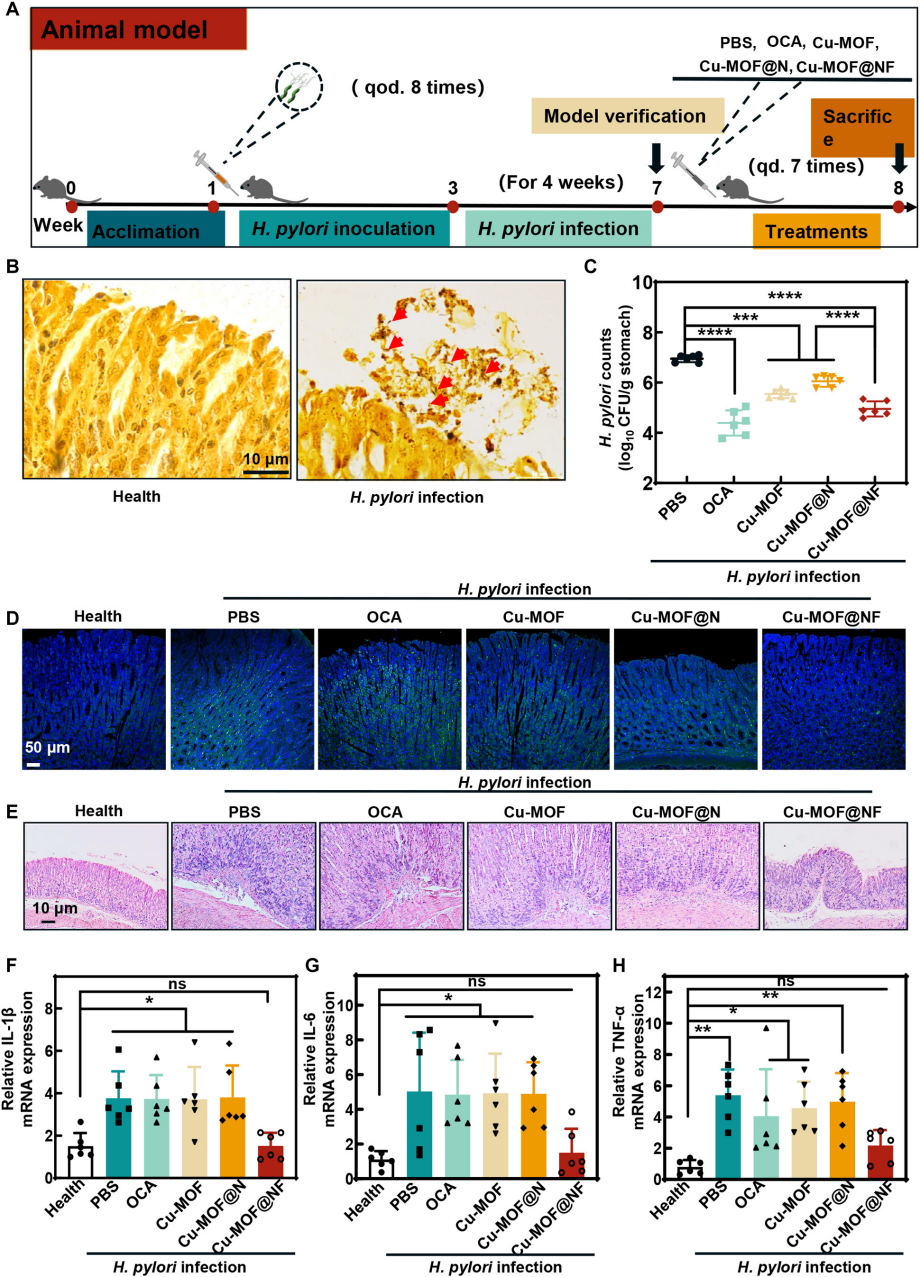
Treatment of *H. pylori* infection in vivo*.* (A) Schematic diagram of modeling and treatment of *H. pylori*-infected mice. (B) Warthin-starry silver staining of the gastric mucosa of mice for *H. pylori* colonization. (C) Quantification of antimicrobial activity of *H. pylori* in vivo. (D) Schematic diagrams of the mucosa of gastric tissues for ROS. (E) H&E staining of the murine stomachs from the different treatment groups. (F to H) RT-qPCR analysis showing mRNA levels of inflammatory cytokines like IL-1β, IL-6, and TNF-α in the gastric tissues of mice in the indicated groups. Data are means ± SD (*n* = 6). ns indicates not significant, with *P* ≥ 0.05. *P < 0.05, **P < 0.01, ***P < 0.001, ****P < 0.0001.

The outstanding therapeutic ability of Cu-MOF@NF in vitro prompted us to explore its efficacy for treating *H. pylori* infection in vivo. C57BL/6 mice were randomized into the healthy group, PBS group, Cu-MOF group, Cu-MOF@N group, Cu-MOF@NF group, and triple therapy (omeprazole, clarithromycin, and amoxicillin [OCA]) group. As shown in Fig. 7A, the healthy group was administered Brucella broth, and the other 5 groups were treated with 1 × 10^8^ CFU ml^−1^ solution of rodent-adapted CagA^+^ *H. pylori* strain PMSS1 once every other day for 8 consecutive times. After 4 weeks of infection, the results of Warthin-starry silver staining, plate coating, and Gram staining showed that *H. pylori* successfully colonized the stomachs of mice (Fig. 7B and Fig. S20). Next, the synthesized materials were administered orally, and the mice were euthanized the following day after drug withdrawal to perform further validation. It was observed that Cu-MOF, Cu-MOF@N, and Cu-MOF@NF all eradicated *H. pylori* in murine stomachs to a certain extent, indicating that Cu-MOF and Cu-MOF@N still had antibacterial properties against floating *H. pylori* in murine stomachs. Cu-MOF@NF had good mucus penetration function and antibacterial effects on both gastric planktonic bacteria and mucosal colonizing bacteria. Therefore, it had the best antimicrobial effect among the 3 groups, second only to OCA (Fig. 7C). Subsequently, ROS fluorescence probe was used to stain the gastric mucosal tissue sections of mice, which was observed and photographed under CLSM. The green fluorescence was positively proportional to the ROS level, and the results showed that the ROS levels of the gastric mucosa in the mice of the Cu-MOF@NF group were significantly lower in comparison with those of the other groups (Fig. 7D). After that, hematoxylin-eosin (H&E) staining and inflammation scoring of mice gastric tissue showed extensive inflammatory cell infiltration and thickened glands beneath the mucus layer of the PBS, Cu-MOF, Cu-MOF@N, and OCA groups, whereas no obvious inflammatory lesions were seen in the Cu-MOF@NF group (Fig. 7E and Fig. S21). The expression of inflammatory cytokines in the gastric tissues of mice was also detected by RT-qPCR, which showed that pro-inflammatory cytokines, including IL-1β, IL-6, and TNF-α, were down-regulated in the Cu-MOF@NF group (Fig. 7F to H). This was attributed to the antioxidant and inflammatory microenvironment improving properties of NGCD released by Cu-MOF@NF after penetrating the mucus layer. However, the antioxidant and improving inflammatory microenvironment effects were not observed in the Cu-MOF@N group due to poor mucus permeability, which prevented the released NGCD from penetrating the mucus layer. The above results indicated that Cu-MOF@NF could regulate the inflammatory response of gastric mucosa. To investigate the biosafety of Cu-MOF@NF, the mice were first monitored for body weight and blood biochemical indices. Blood biochemical indices were tested after 7 days of treatment, including creatinine (Crea) and urea (Urea) reflecting renal function, aspartate aminotransferase, alanine aminotransferase, and alkaline phosphatase reflecting hepatic function (Fig. S22). In addition, no significant difference in body weight was observed before and after treatment in each group of mice (Fig. S23). In addition, the in vivo histotoxicity of Cu-MOF@NF was determined by histological analysis of major organs (heart, liver, spleen, lung, and kidney). Based on the H&E staining results of Fig. S24, no obvious histological abnormalities or inflammatory lesions were observed in the major organs of the mice of the Cu-MOF@NF group. This indicated that Cu-MOF@NF had no obvious toxic side effects on mice after a short-term 7-day treatment. It was confirmed that almost all of the material were emptied in the mice after 24 h of oral administration (Fig. S25). Aiming at investigating the long-term biocompatibility of Cu-MOF@NF in mice, the concentration of Cu^2+^ in the murine important organs (heart, liver, spleen, lung, kidney, stomach, and intestine) was detected after the Cu-MOF@NF was continuously administered to mice by gavage for 1 month. The Cu^2+^ content in the important organs treated with Cu-MOF@NF was similar to that of the healthy mice, indicating negligible accumulation of Cu-MOF@NF (Fig. S26).

### Investigation on the protection of gut flora

**Fig. 8.**
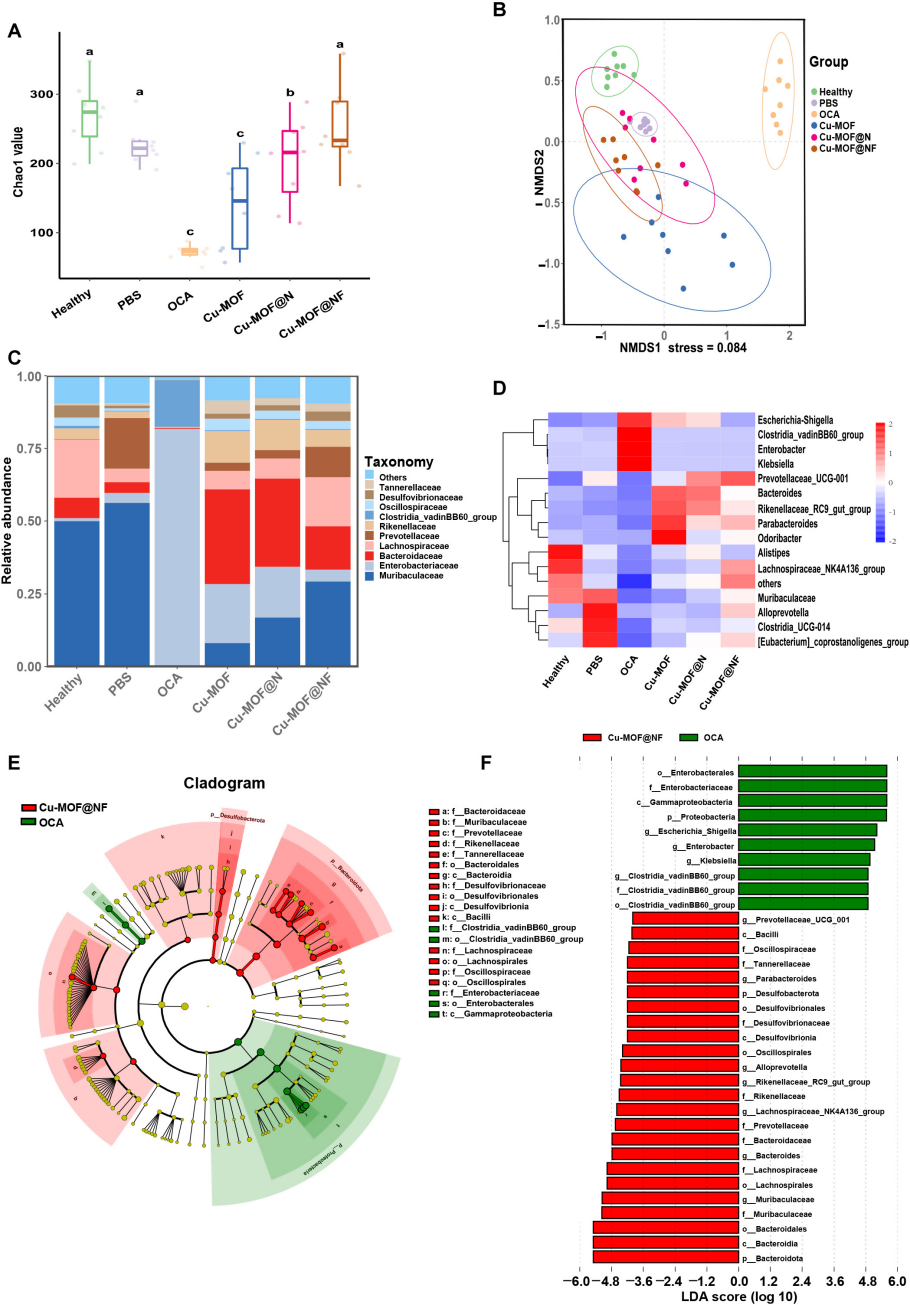
Conservation of gut flora by Cu-MOF@NF. (A) Gut flora abundance Chao1. Compact letter displays (CLDs) were used if the logarithm of the difference grouping was greater than 5, and 2 groups sharing one or more letters indicated that they were not “significantly” different. (B) NMDS plot showing the β-diversity of the gut microbiota. (C) The relative abundance histogram of gut microbiota at the family level. (D) Heatmap of relative abundance of the top 15 observed operational taxonomic units at the genus level. (E) Evolutionary meristems radiating in a circle from the inside to the outside represented the taxonomic hierarchy from phylum to species. Red nodes in the branches signified microbial taxa that played a vital role in the Cu-MOF@NF group, and green nodes signified microbial taxa that played a key role in the OCA group. The species names indicated by letters in the figure were labeled with the legend at the upper right corner. (F) Green and red regions of the LDA value distribution histogram indicated different subgroups, where the red nodes of the branches showed microbial taxa significant among the Cu-MOF@NF group, and green nodes showed microbial taxa significant among the OCA group. It was listed for all species whose LDA scores exceeded 3.6. The species names indicated by letters in the figure were labeled at the upper right corner.

The dynamic imbalance of intestinal flora induced by conventional antibiotic therapy results in diverse enteric disorders. Accordingly, feces were collected from each group of mice after treatment, and 16S rRNA sequencing was utilized to assess the abundance, variety, and community architecture of gut flora. The α-diversity (Chao1) of the Cu-MOF@NF group showed little marked difference in comparison with that of mice in the healthy group, whereas there was a notable alteration in the OCA group (Fig. 8A). The principal coordinates analysis was performed to assess the overall differences in β-diversity. Non-metric multidimensional scaling (NMDS) plots revealed that the OCA group and the healthy group were clustered separately. However, the gut microbiota of the Cu-MOF@NF group was closer to that of the healthy group (Fig. 8B). These data indicated that Cu-MOF@NF had minimal impact on the overall microbial abundance of the gut microbiota. Subsequently, an in-depth analysis of the gut microflora was conducted. The OCA group showed a striking increase in the relative abundance of pathogenic bacteria within the family level, such as *Enterobacteriaceae* (deleterious to homeostasis in the gut) [53]. On the other hand, in the OCA group, the relative abundance of beneficial bacteria, including *Muribaculaceae* and *Lachnospiraceae* (which alleviate gut inflammation, improve gut energetic metabolism, maintain enteric mucosal intactness, and stabilize the immune status through the generation of short-chain fatty acids) [54,55], was drastically decreased. Nevertheless, the relative abundance of the Cu-MOF@NF group showed little marked variation from the healthy group (Fig. 8C). Notably, the Cu-MOF@NF group significantly reduced the relative abundance of *Escherichia, Shigella*, *Enterobacter*, and *Klebsiella* (noted to generate extracellular toxins and trigger enteric inflammation) [56] compared with the OCA group (Fig. 8D). Linear discriminant analysis (LDA) effect size (LEfSe) analysis was used to characterize specific microbiota across the Cu-MOF@NF and OCA groups. Notable variations in the gut microbiota were observed across the 2 groups. The OCA group harbored increased harmful bacteria including *Bacilli*, *Enterobacteriaceae*, and *Aspergillus*, whereas a variety of probiotic bacteria, including *Muribaculaceae* and *Lachnospiraceae*, were the major contributors in the Cu-MOF@NF group (Fig. 8E and F). In other words, after OCA exposure, beneficial bacteria were inhibited, and harmful bacteria were provided with advantageous survival and proliferation conditions. Nevertheless, the Cu-MOF@NF was critical to preserve the gut microecological balance because it had no antimicrobial effect in the gut, providing advantageous conditions for the survival and proliferation of beneficial bacteria. To sum up, Cu-MOF@NF had no significant effect on the abundance, function, and species variety of the commensal gut microbiota and exerted an important contribution to the protection of the intestinal microecological balance, which might potentially serve as an effective treatment of *H. pylori* infection without causing intestinal diseases.

## Conclusion

A Cu-MOF platform, Cu-MOF@NF, with mucus-penetrating function has been developed to treat *H. pylori* infection. The system can penetrate the mucus layer and efficiently block the periodic growth pattern of “planktonic bacteria ↔ biofilm”, thus reducing the incidence of recurrent infections. In contrast to traditional triple therapy, Cu-MOF@NF can exhibit antibacterial properties and improves the inflammatory microenvironment without disrupting the balance of intestinal flora. However, the platform has not yet been systematic compared with antibiotics through different experiments, and its inhibitory effect on *H. pylori* biofilm and its ability to specifically target *H. pylori* require further investigation. This work provides a strategy to construct antibiotic-free platform that can penetrate mucus and have antibacterial effects and improve the inflammatory microenvironment. This may open up new paradigms for treating respiratory diseases and other gastric intestinal-related diseases characterized by the presence of a physical mucus barrier.

## Materials and Methods

### Materials

Copper chloride dihydrate (CuCl_2_·2H_2_O, 99%), polyvinylpyrrolidone (PVP, Mw 30,000), L-ascorbic acid (99%), sodium hydroxide (NaOH, 97%), D (+)-anhydrous glucose (98%), urea (99%), and LPS were derived from Shanghai Macklin Biochemical Co., Ltd. (China). Benzenedicarboxylic acids (H_2_BDC, 98%), *N*,*N*-dimethylformamide (DMF, 99%), amoxicillin sodium (89%), clarithromycin (99%), omeprazole (98%), methanol (99%), and ethanol (99%) were purchased from Aladdin Biochemical Technology Co., Ltd. (China). FU (UV ≥ 90%), Warthin-starry silver staining solution, ROS assay kit, ATP content assay kit, and β-galactosidase (β-GAL) activity assay kit were bought from Beijing Solarbio Science & Technology Co., Ltd. (China). TRIZOL reagent was purchased from Beijing Tianen Biotechnology (China). The ROS probe and monoclonal antibody against MUC5AC were purchased from Thermo Fisher Scientific. Wild-type ATCC 43504 and the rodent-adapted strain CagA^+^ *H. pylori* pro-mouse Sydney strain 1 (PMSS1) were utilized to conduct this research. The gastric mucosa cell line (HFE145) and human gastric epithelium (GES-1) cells were cultured in Dulbecco’s modified Eagle’s medium (DMEM) and Roswell Park Memorial Institute (RPMI/1640) culture medium containing 10% fetal bovine serum (FBS, Gibco, Carlsbad, CA) and 1% penicillin/streptomycin (Gibco, Carlsbad, CA), respectively. Brucella broth (BD) and brain heart infusion (BHI) were obtained from Bioscience (Australia). High-purity water (Millipore Milli-Q grade) with a resistivity of 18.2 MΩ was used in all the experiments. All chemical reagents used were of analytical grade and were not further purified.

### Sample preparation

#### Synthesis of cu-MOF nanosheets

First, Cu-MOF nanosheets were preliminarily synthesized using previous reports [57]. Briefly, CuCl_2_·2H_2_O (0.1 mmol) and PVP (0.1 g) were dissolved in water (40 ml), and then NaOH (2.5 ml, 0.2 M) was added slowly and dropwise. The mixture was under magnetic stirring for 5 min, followed by the slow dropwise addition of L-ascorbic acid (2.5 ml, 0.1 M). Upon the addition of L-ascorbic acid, the mixture was stirred for another 5 min and washed 3 times with ethanol. Subsequently, the yellow liquid was redispersed in ethanol (10 ml) for future use. H_2_BDC (0.5 mmol) was completely dissolved in ethanol (5 ml). Then, DMF (5 ml) and the yellow liquid (ethanol, 10 ml) as prepared above were added. Subsequently, the reaction was fully contacted with oxygen at room temperature for 5 h, so that the yellow liquid gradually turned into a blue liquid. Finally, the blue products were centrifuged for collection, which were washed 3 times by methanol and ethanol respectively, and freeze-dried to obtain Cu-MOF nanosheets.

#### Synthesis of NGCD

Glucose (1 g) and urea (0.5 g) dissolved in deionized water (60 ml) agitatedly were transferred to a polytetrafluoroethylene-lined stainless steel reactor (100 ml) to react at 200 °C for 6 h. Following the cool-down, the reaction product was centrifuged at 10,000 rpm for 30 min. The supernatant was then filtered through a nylon filter with a pore size of 0.22 μM. The black NGCD powder was obtained by freeze-drying.

#### Synthesis of cu-MOF@N

Cu-MOF (5 mg) was dispersed in ethanol solution (10 ml), and then ethanol solution (10 ml) containing NGCD (10 mg) was added, followed by magnetically stirring at room temperature for 1 h. The solution was sonicated in the dark with the ultrasonic cleaner (40 kHz) for 20 min and stirred overnight. After that, Cu-MOF@N was collected by centrifugation, eluted with ethanol 3 times, and freeze-dried.

#### Synthesis of cu-MOF@NF

The synthesized Cu-MOF@N nanoparticles (3 mg) were dispersed in ethanol (10 ml). After that, FU in ethanol (1 ml) was sonicated with ultrasonic cleaner (40 kHz) in the dark for 30 min, added to the suspension of Cu-MOF@N nanoparticles, and magnetically stirred at room temperature away from light for 24 h. The solution was washed 3 times by ethanol and finally freeze-dried to obtain Cu-MOF@NF.

### Mucus penetration test

FITC was used to label NGCD, FU, Cu-MOF, Cu-MOF@N, and Cu-MOF@NF, respectively. Hyaluronic acid (1.2%) and 0.2% gastric mucin were chosen to prepare gastric mucus mimics. Firstly, PBS (1 ml) solution was added to the lower layer of the 24-well transwell plate, and gastric mucus mimic (100 μl) was added to the upper chamber to make it lay flat on the membrane of the upper chamber. Another 100 μl of FITC-labeled NGCD, FU, Cu-MOF, Cu-MOF@N, and Cu-MOF@NF was added, and the transwell plate was placed in a shaker (37 °C, 100 rpm) and shaken for 4 h. Finally, the solution in the lower chamber was aspirated, and the light spectrum of the solution was measured by a fluorospectrophotometer. To examine the mucus penetration ability of the materials, NGCD, FU, Cu-MOF, Cu-MOF@N, and Cu-MOF@NF were placed on hardened gelatin gels [58,59]. The samples were stained with Coomassie brilliant blue and deposited on the mucus layer of the gastric mucus mimic. Images of the samples at 0 min, 30 min, and 60 min were taken and recorded.

### Inhibition of *H. pylori* adhesion to gastric epithelial cells

The *H. pylori* PMSS1 strain was cultured overnight in BD supplemented with 10% FBS, and HFE145 cells at a density of 1 × 10^5^ cells per well in 20-mm-diameter crawler sheets were cultured overnight until adherence, followed by adding DMEM containing 25 μg ml^−1^ Cu-MOF@NF and incubation for 2 h. Subsequently, bacteria were co-incubated with HFE145 cells for 6 h with a 100:1 multiplicity of infection (MOI). After washing with cold PBS, cells were fixed in 4% formaldehyde with 30 min. Thereafter, they were permeabilized in 0.3% Triton X-100 at room temperature with 15 min, blocked by 3% bovine serum albumin (BSA) with 1 h, washed 3 times with PBS, and then incubated using *H. pylori* antibody at 4 °C overnight. On the second day, cells were washed 3 times and incubated using Alexa Fluor Plus555 (1:500, Thermo Fisher) secondary antibody for 1 h away from light. Finally, cell nuclei were labeled with Hoechst 33342 and imaged with the CLSM (Leica Stellaris5).

### *H. pylori* culture and in vitro antimicrobial test

*H. pylori* was cultured in Brucella agar with 5% sheep blood in a 3-gas incubator (37 °C, 5% O_2_, 10% CO_2_). Bacteria in Brucella agar were scraped off, suspended in BHI, and centrifuged at 4,000 rpm to adjust the density to 1 × 10^8^ CFU ml^−1^. Cu-MOF@NF nanoparticles with different concentrations (120, 150, 180, and 200 μg ml^−1^) were placed into BHI culture solution (10 ml) containing 10% FBS in culture flasks and incubated in a 3-gas incubator for 12 h. The mixtures were further diluted by pipetting in 50 μl of diluent and applied on co-cultivated blood plates for 72 h to perform colony counting on the plates.

### Observation of bacterial morphology with SEM and live/dead staining experiments

Bacteria were co-cultured with the material, centrifuged, washed 3 times with PBS, and fixed by 2.5% glutaraldehyde for 2 h. These mixtures were subsequently dehydrated in different concentrations of ethanol solutions and finally dispersed in tert-butanol. The solution was dropped on silicon wafers for freeze-drying, sprayed with gold, and visualized with SEM. For live/dead bacteria staining, specimen-treated *H. pylori* solution was incubated with SYTO 9/PI staining solution for 15 min and imaged by inverted fluorescence microscopy.

### Antibacterial mechanism experiment

(a) Cell membrane permeability of *H. pylori*. The permeability was evaluated with a β-galactosidase kit. Bacterial supernatants containing different materials were co-incubated with the test solution for 30 min, and the optical density (OD) of the test solution at 420 nm was determined using the enzyme plate analyzer. (b) ATP level detection. Bacterial supernatants co-cultured with different materials were assayed for the amount of ATP using an ATP assay kit.

### Biofilm destruction experiments

First, strain ATCC 43504 in Brucella agar was scraped off and suspended in BHI. It was fully blown and mixed to adjust the density to 1 × 10^8^ CFU ml^−1^ for backup. Then, the cover glass was placed at the bottom of each well in the 6-well plate, and 2 ml of the above *H. pylori* suspension was added to each well, which was incubated for 72 h to allow it to form a biofilm on the cover glass. Subsequently, the supernatant was carefully removed, and 180 μg ml^−1^ FU, NGCD, Cu-MOF, Cu-MOF@N, and Cu-MOF@NF nanoparticles was added to be co-incubated together for 24 h. Thereafter, the supernatant was discarded. The cover glass was washed and then stained by SYTO 9 dye at 37 °C for 30 min. The cover glass with the biofilm attached was then taken out onto slides, sealed, and imaged with CLSM to obtain the 3D structure of the biofilm.

### Quantification of live bacteria in biofilm

After the biofilms were co-incubated with the nanomaterials by the above methods, the insulin needle was used to remove the bacterial fluid. The biofilm was carefully and gently washed 2 to 3 times with PBS. Subsequently, the biofilm was gently rinsed with PBS for 2 to 3 times, and then the biofilm was collected and treated with an ultrasonic generator for 10 min to disperse it. The suspension was serially diluted, evenly coated on the BD plate (100 μl in total), and cultured inverted in the incubator at 37 °C for 24 h. Eventually, counts of plate colonies were made.

### Biofilm inhibition experiment

At first, the ATCC 43504 strain in Brucella agar was scraped off and suspended in the BHI by blowing and mixing thoroughly to adjust the density to 1 × 10^8^ CFU ml^−1^ for future use. Subsequently, slides were placed at the bottom of each well in the 6-well plate, and 2 ml of the above-mentioned *H. pylori* suspension was added to each well, while 180 μg ml^−1^ FU, NGCD, Cu-MOF, Cu-MOF@N, and Cu-MOF@NF nanoparticles was added and co-incubated together for 72 h to allow it to form biofilm on the slides. The biomass was later measured using crystal violet staining.

### Electron paramagnetic resonance capture of free radicals experiment

Removal of superoxide anion free radicals (O_2_^•**−**^**)** by Cu-MOF@NF was determined using an ESR spectrometer with a microwave power of 0.6 mW and 1-G modulation amplitude. Potassium superoxide (KO_2_) and 5-tert-butoxycarbonyl-5-methyl-1-pyrroline N-oxide (BMPO) served as the O_2_^•**−**^ promoter and spin trapping agent. The mixture was composed of 2.5 mM KO_2_, 25 mM BMPO, and 3.5 mM 18-crown-6 in dimethyl sulfoxide (DMSO) medium. ESR spectra were obtained in the presence or absence of Cu-MOF@NF (180 μg ml^−1^).

For the scavenging of hydroxyl radical (^•^OH) by Cu-MOF@NF, we used ESR spectroscopy in the setup as aforementioned. ^•^OH was produced by UV irradiation of 5 mM hydrogen peroxide in 10 mM buffer for 5 min and captured by 50 mM BMPO. ESR spectra were obtained in the presence or absence of Cu-MOF@NF (180 μg ml^−1^).

For nitric oxide free radicals (^•^NO) scavenging by Cu-MOF@NF, 250 μM S-nitroso-N-acetylpenicillamine (SNAP) was applied for a source of ^•^NO, and ESR spectroscopy was exploited in the above setup. The capture of ^•^NO with Carboxy-PTIO (10 μM) of the 5-line electron paramagnetic resonance signal yielded carboxy-p-cresol (CpI) of the 7-line electron paramagnetic resonance signal. ESR spectra were recorded in the presence or absence of Cu-MOF@NF (180 μg ml^−1^).

### In vitro intracellular clearance of ROS

To validate the ability of gastric mucosal epithelial cells to clear intracellular ROS, HFE145 cells (8,000 cells per well) were incubated in 96-well plates with DMEM containing Cu-MOF@NF (180 μg ml^−1^) for 6 h. Diluted ROS up solution was added after removing the medium. After 1 h, the mixture of DCFH-DA and Hoechst 33342 was added for 30 min. The cells were washed twice by PBS and imaged under the fluorescence microscope.

### Quantitative reverse transcription polymerase chain reaction analysis

HFE145 cells (6,000 cells per well) were inoculated in 6-well plates overnight. Subsequently, the LPS and Cu-MOF@NF groups were treated with LPS (1 μg ml^−1^) for 4 h and washed 3 times with PBS, respectively. The control and LPS groups were then replaced with a complete medium, and the Cu-MOF@NF group was replaced with a medium mixture containing Cu-MOF@NF (180 μg ml^−1^) for 6 h. Following this, whole RNA was extracted with TRIZOL reagent. Primers are listed in Table S2.

### Hemolysis test

Fresh blood from mice was centrifuged at 1,500 rpm to obtain a precipitate of red blood cells (RBCs), which was diluted with saline. RBCs (100 μl) and 100 μl of Cu-MOF@NF (25, 50, 100, and 200 μg ml^−1^) solution were added to 1 ml of saline. In parallel, 100 μl of erythrocytes in saline (1.1 ml) and deionized water (1.1 ml) were taken as negative and positive control, respectively. Then, after the water bath at 37 °C for 2 h, the absorbance value 540 nm was measured by an enzyme-labeled instrument. The hemolytic rate was calculated as follows:

$$\text{Hemolysis rate}\;\left(\%\right)\;=\;\left({\text{OD}}_{0}-{\text{OD}}_{1}\right)/\left({\text{OD}}_{2}-{\text{OD}}_{1}\right)\times 100\%$$ (1)

where OD_0_ stands for experimental group values, OD_1_ denotes negative control group values, and OD_2_ indicates positive control group values.

### Cytocompatibility test

HFE145 cells were cultured in DMEM containing 10% FBS and 1% penicillin for 24 h at 37 °C in a 5% CO_2_ incubator, and then inoculated in 96-well plates (2,000 cells per well) for another 24 h of incubation. Subsequently, the medium mixture (100 μl) containing different concentrations of Cu-MOF@NF was added and the cells were incubated for 1 day and 2 days. After this, 20 μl of CCK-8 solution was added, and the cells were incubated for 2 h away from light before the absorbance was measured by the multifunctional enzyme-labeled instrument. Cell viability was measured by the following formula:

$$\text{Cell viability}\;\left(\%\right)=\left({\text{OD}}_{a}-{\text{OD}}_{b}\right)/\left({\text{OD}}_{c}-{\text{OD}}_{b}\right)\times 100\%$$ (2)

where OD_a_ stands for the value of the experimental groups, OD_b_ denotes the value of the blank group, and OD_c_ indicates the value of the control group.

### Live/dead cell staining experiment

HFE145 cells (10,000 cells per well) were incubated in 24-well culture plates for 24 h. The medium was in turn switched to fresh medium (1 ml) with different concentrations of Cu-MOF@NF, and the cells were incubated for another 24 h. After that, the cells were stained with calcein-AM and propidium iodide (PI) dyes and imaged using inverted fluorescence microscopy.

### In vivo therapeutic experiments

All the in vivo studies complied with institutional guidelines for animal care and underwent approval by the animal ethics committee of Nanchang University (Nanchang, China, NCULAE-20221228028). First, *H. pylori*-infected mice models were constructed. C57BL/6 mice were randomized into 6 groups (healthy group, PBS group, Cu-MOF group, Cu-MOF@N group, Cu-MOF@NF group, and triple therapy [OCA] group), with 6 mice in each group. The healthy group was given Brewer’s broth by gavage, and the remaining 5 groups received oral administration of the 1 × 10^8^ CFU ml^−1^ solution containing rodent-adapted strain CagA^+^ *H. pylori* Pre-Mouse Sydney Strain 1 (PMSS1) once every other day for 8 consecutive times. For 4 weeks after the mice were infected, the gastric infection was detected by Warthin-starry silver staining, plate coating, H&E staining, and Gram staining. After confirming the colonization, the infected C57BL/6 mice were randomized into 6 groups (*n* = 6 each group), composed of the healthy group, PBS group, Cu-MOF group, Cu-MOF@N group, Cu-MOF@NF group, and OCA group. Subsequently, the corresponding 16 mg kg^−1^ nanomaterials were given to the corresponding nanomaterial group, and the OCA group was given the triple drug (400 μmol kg^−1^ of omeprazole [a proton pump inhibitor], 14.3 mg kg^−1^ of clarithromycin, and 28.5 mg kg^−1^ of amoxicillin) by gastric gavage for 7 days [60]. Mice were executed the day after the last treatment for subsequent biochemical experiments. Gastric tissues were homogenized, diluted, and spread on plates to evaluate the in vivo antimicrobial activity. Formaldehyde-fixed gastric tissue specimens were dehydrated and paraffin-embedded for histological staining analysis. Tissue sections (5 μm thick) were acquired with a sectioning machine to perform H&E staining. The serum biochemical indices analysis was utilized to detect the liver and kidney function of mice. The serum collected from mice was tested in the Laboratory Department of the First Affiliated Hospital of Nanchang University.

### Detection of ROS levels in mouse gastric mucosa tissue

After the wax block section of mouse gastric mucosa tissue was dewaxed, it was washed 3 times by PBS. After gently shaking to discard excess liquid, circles were drawn around the tissues using an immunohistochemical oil pen, and the ROS probe was added dropwise to fully cover the tissues, which were incubated at 37 °C for 30 min, avoiding light. After incubation for 30 min, PBS was used to wash 3 times, each time for 5 min. After gently shaking to remove excess liquid, DAPI staining solution was added and incubated for 20 min at room temperature avoiding light. After this, the slices were sealed with an anti-fluorescence quenching sealer and imaged with CLSM.

### Gut microbiota analysis

After the last treatment, the feces of mice were collected. The abundance and diversity of bacteria and the relative abundance of colony structure in the samples were determined via 16S rRNA sequencing by OE Biotech. Co., Ltd. (China).

### Statistical analysis

Data were analyzed on SPSS 19.0, evaluated as means ± standard deviation based on at least 3 tests, and contrasted via one-way analysis of variance. A probability value (*P* value) below 0.05 was considered statistically significant (*P < 0.05, **P < 0.01, ***P < 0.001, ****P < 0.0001).

## Data Availability

The data that support the findings of this study are available from the corresponding authors upon reasonable request.
