# Supplementary material for: Copper-Bearing Metal-Organic Framework with Mucus-Penetrating Function for the Multi-Effective Clearance of Mucosal Colonized Helicobacter pylori
Source: Research (Wash D C). 2024 May 22;7:0358. doi: 10.34133/research.0358 (PMC11109517; doi:10.34133/research.0358)
Supplement: Supplementary 1 — Figs. S1 to S26 Tables S1 and S2 [file research.0358.f1.docx]

Supplementary Material

**
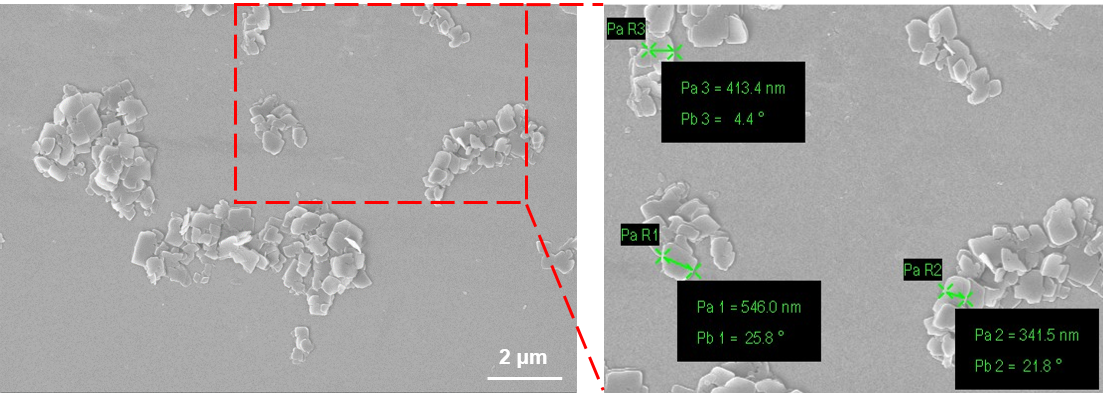
**

**Figure S1.** SEM images of synthesized Cu-MOF.


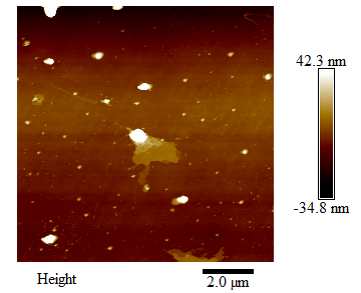


**Figure S2.** Two-dimensional height AFM image (2 µm × 2 µm) of several Cu-MOF nanosheets (where the thickness data were obtained by measuring the height profile along the trajectory shown in the micrograph).


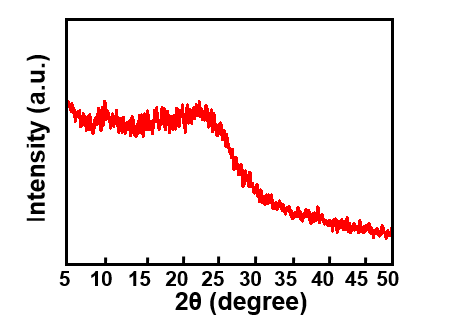


**Figure S3.** XRD spectrum of NGCD.


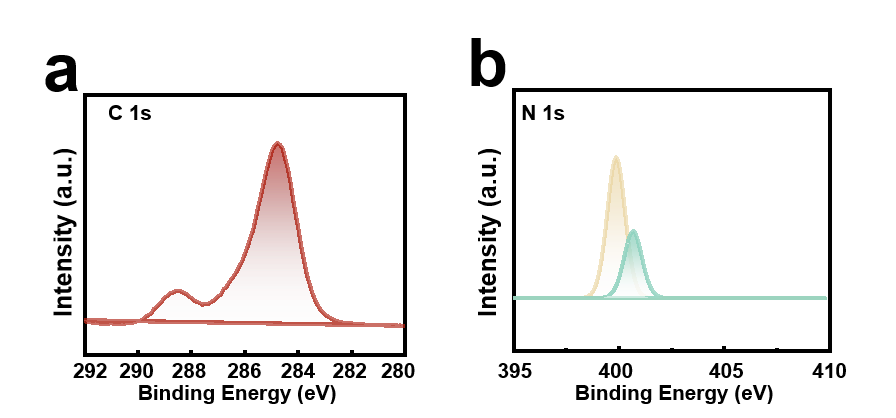


**Figure S4.** High-resolution XPS spectra of C 1s and N 1s in Cu-MOF@N.


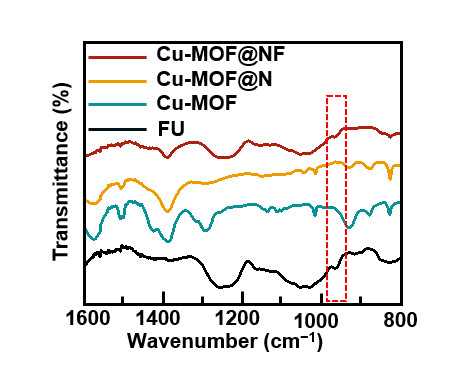


**Figure S5.** Partially magnified FTIR spectra of Figure 1h.


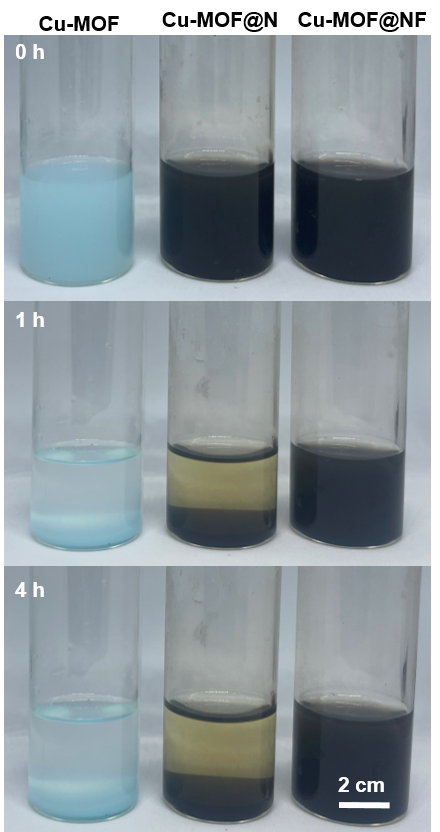


**Figure S6.** Long-term detection of Cu-MOF, Cu-MOF@N, and Cu-MOF@NF in simulated gastric juice.


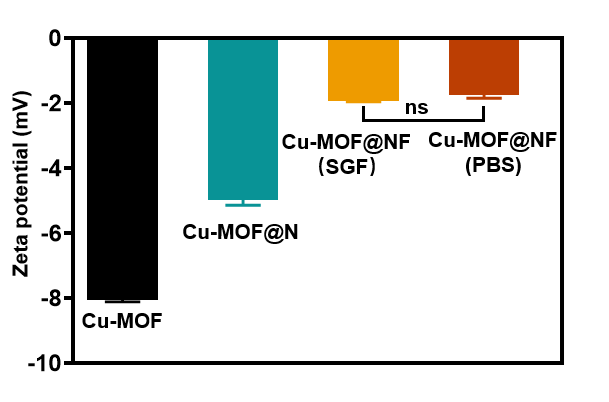


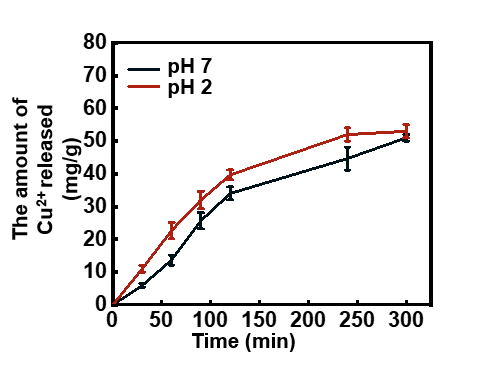
**Figure S7.** Zeta potential diagrams of Cu-MOF, Cu-MOF@N, and Cu-MOF@NF within the simulated gastric fluid (SGF) and Cu-MOF@NF within PBS. Data are means ± s.d. (n ≥ 3).8

**Figure S8.** In vitro release curves of Cu^2+^ in solutions of different pH values. Data are means ± s.d. (n = 3).


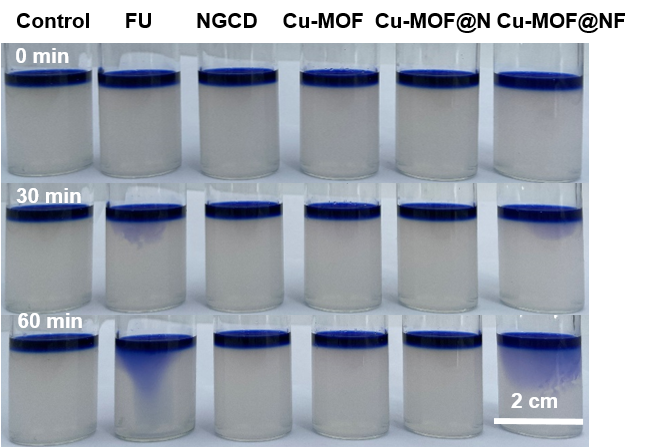


**Figure S9.** Images of nanoparticle products penetrating agar *in vitro*.


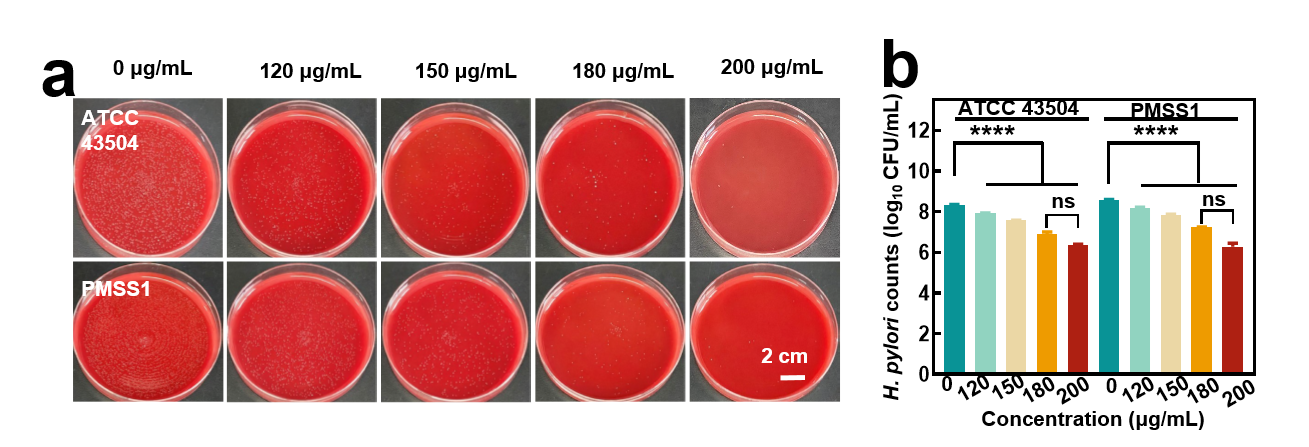


**Figure S10.** a) Plate diagrams of *H. pylori* colonies after treatment with different concentrations of Cu-MOF@NF in *H. pylori* strains ATCC 43504 and PMSS1, respectively. b) Quantification of antibacterial activity in Figure S10a). Data are means ± s.d. (n ≥ 3). Ns indicates not significant, with *p* ≥ 0.05. **p* < 0.05, ***p* < 0.01, ****p* < 0.001, *****p* < 0.0001.


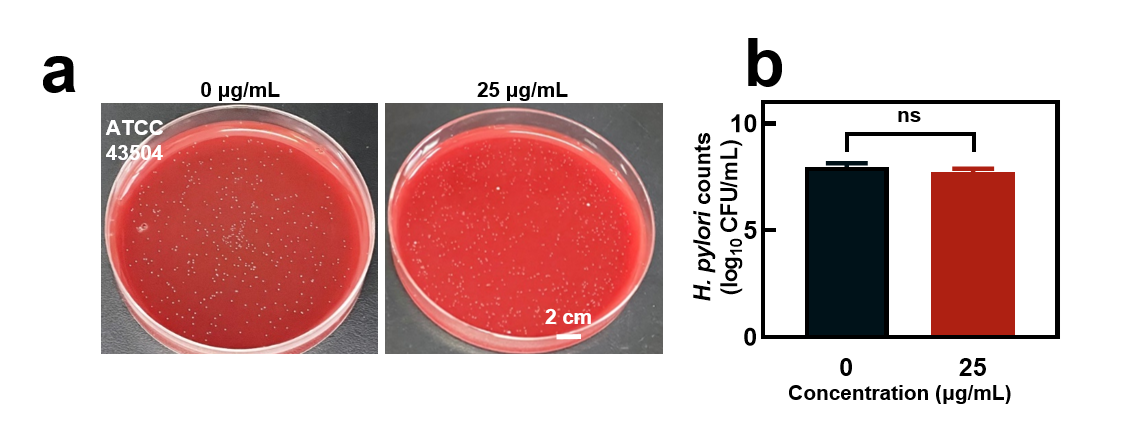


**Figure S11.** a) Colony plate plots of *H. pylori* ATCC 43504 colonies after treatment with 25 μg mL^−1^ concentration of Cu-MOF@NF. b) Antimicrobial activity was quantified in Figure S11a). Data are means ± s.d. (n ≥ 3). Ns indicates not significant, with *p* ≥ 0.05. **p* < 0.05, ***p* < 0.01, ****p* < 0.001, *****p* < 0.0001.


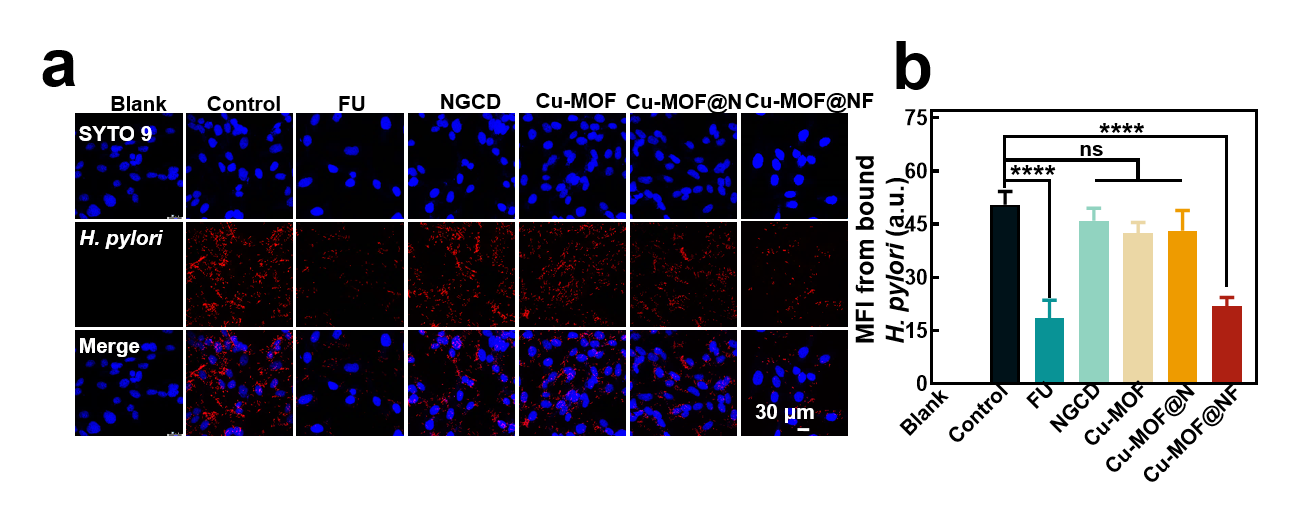


**Figure S12.** a) Fluorescence images of the binding of *H. pylori* to GES-1 cells after each group of GES-1 cells were incubated with the nanoparticles first and then treated with the addition of *H. pylori.* b) Quantitative analysis of the mean fluorescence intensity of *H. pylori* in Figure S12a. Data are means ± s.d. (n ≥ 3). Ns indicates not significant, with *p* ≥ 0.05. **p* < 0.05, ***p* < 0.01, ****p* < 0.001, *****p* < 0.0001.


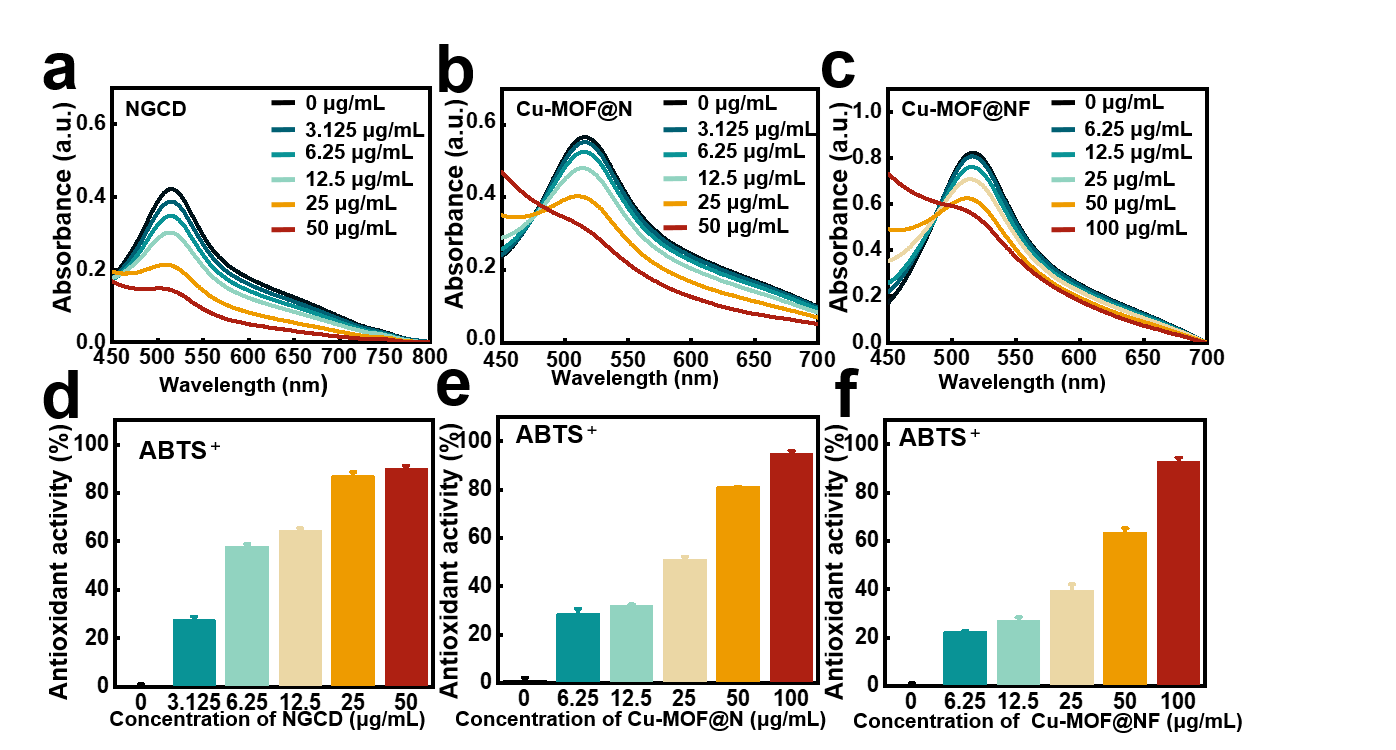


**Figure S13.** Antioxidant activity (DPPH scavenging assay and ABTS) of the NGCD, Cu-MOF@N, Cu-MOF@NF at different concentrations. Data are means ± s.d. (n ≥ 3).
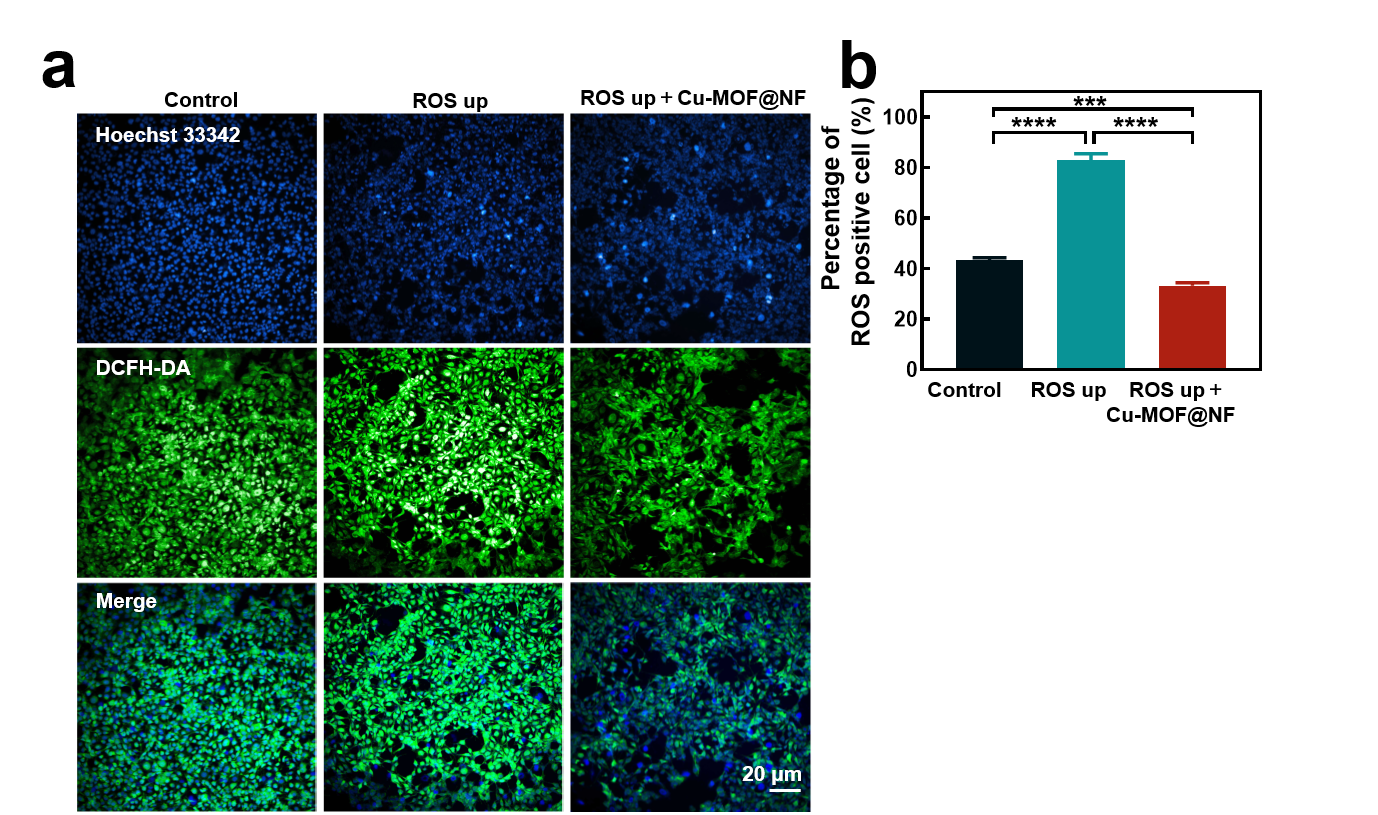


**Figure S14.** a) Intracellular ROS levels in GES-1 cells after treatment with Cu-MOF@NF (180 μg mL^−1^). b) Quantitative statistical plots of DCFH-DA in Figure S13a). Data are means ± s.d. (n ≥ 3). **p* < 0.05, ***p* < 0.01, ****p* < 0.001, *****p* < 0.0001.


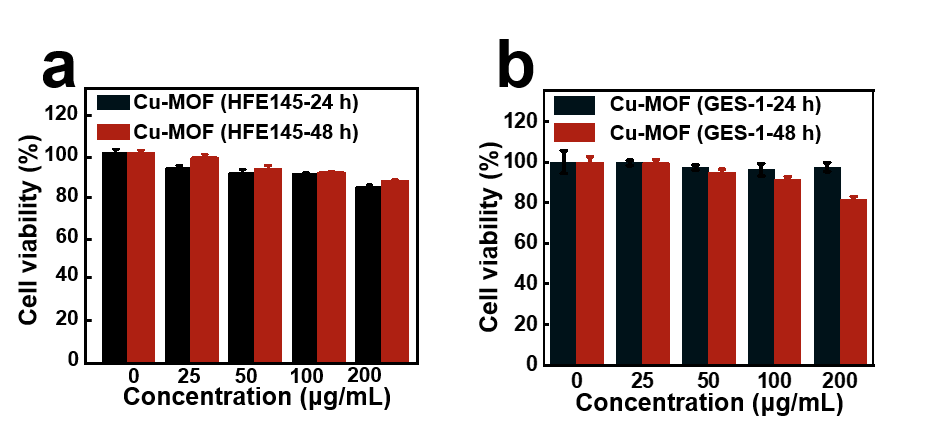


**Figure S15.** Cytotoxicity evaluation of Cu-MOF on HFE145 cells (a) and GES-1 cells (b) for 24 h and 48 h. Data are means ± s.d. (n ≥ 3).


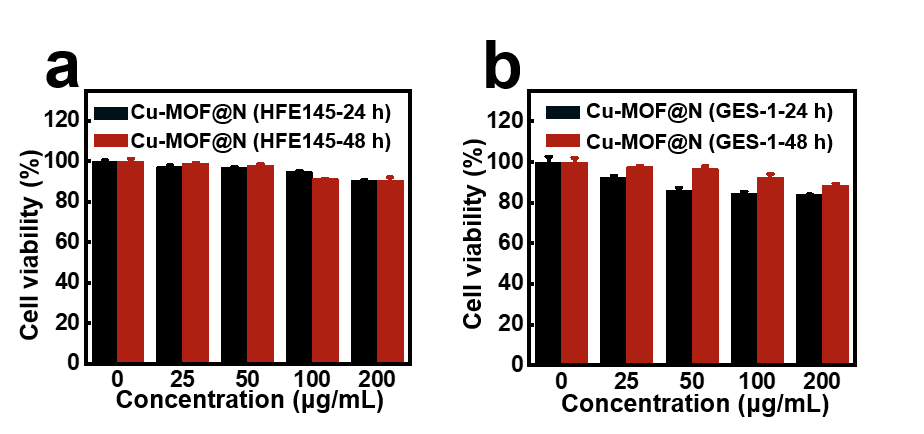


**Figure S16.** Cytotoxicity evaluation of Cu-MOF@N on HFE145 cells (a) and GES-1 cells (b) for 24 h and 48 h. Data are means ± s.d. (n ≥ 3).


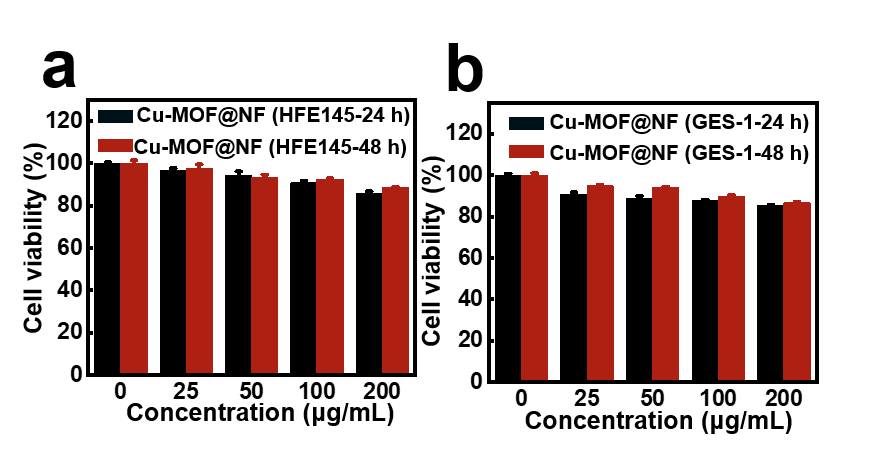


**Figure S17.** Cytotoxicity evaluation of Cu-MOF@NF on HFE145 cells (a) and GES-1 cells (b) for 24 h and 48 h. Data are means ± s.d. (n ≥ 3).


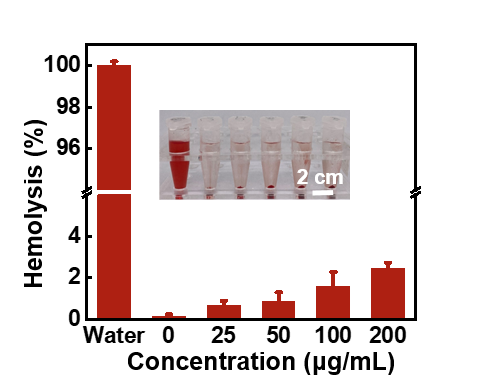


**Figure S18.** Hemolysis analysis of Cu-MOF@NF. Data are means ± s.d. (n ≥ 3).


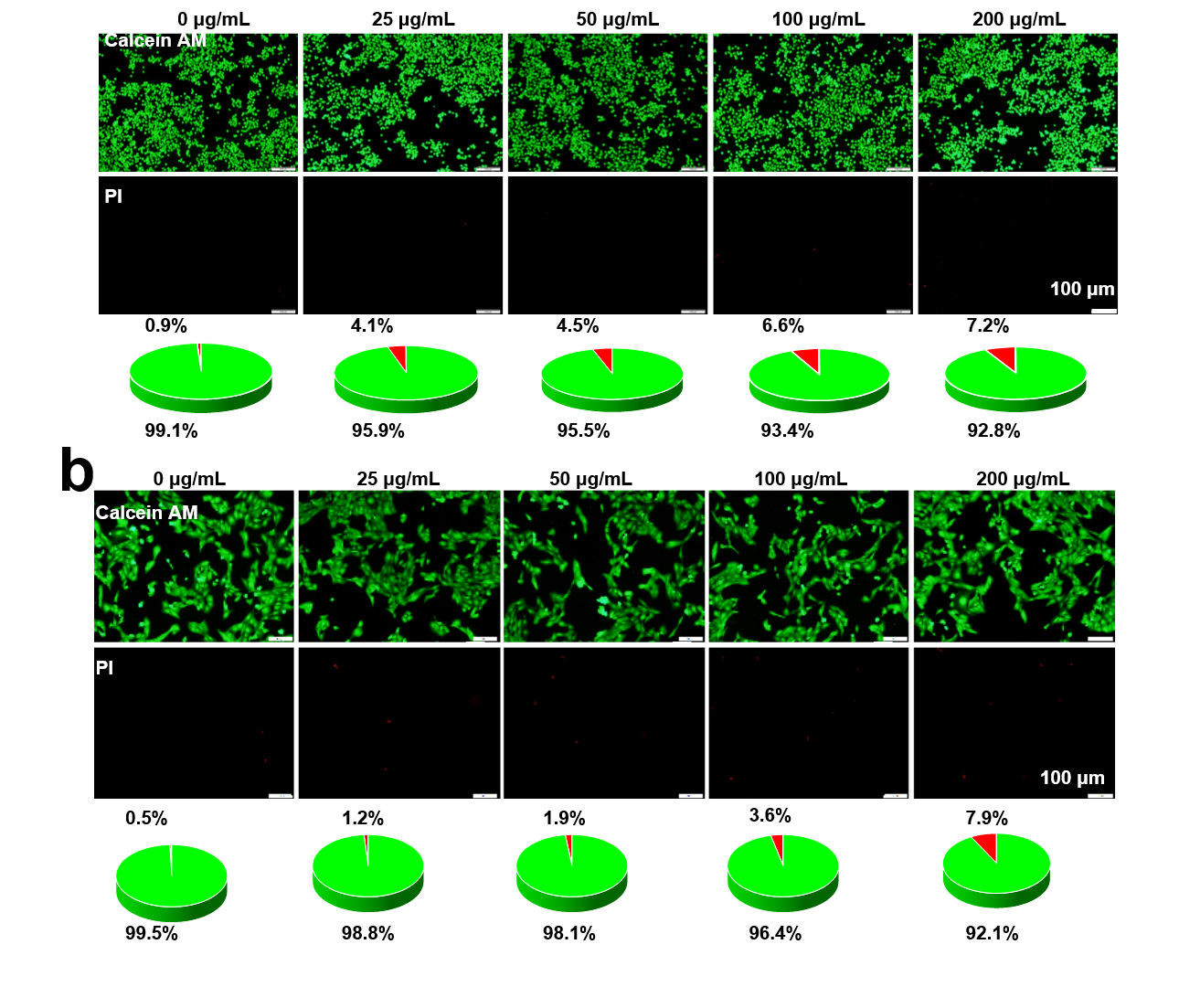


**Figure S19.** Live/dead cell staining of HFE145 cells (a) and GES-1 cells (b) co-cultured with different concentrations of Cu-MOF@NF.


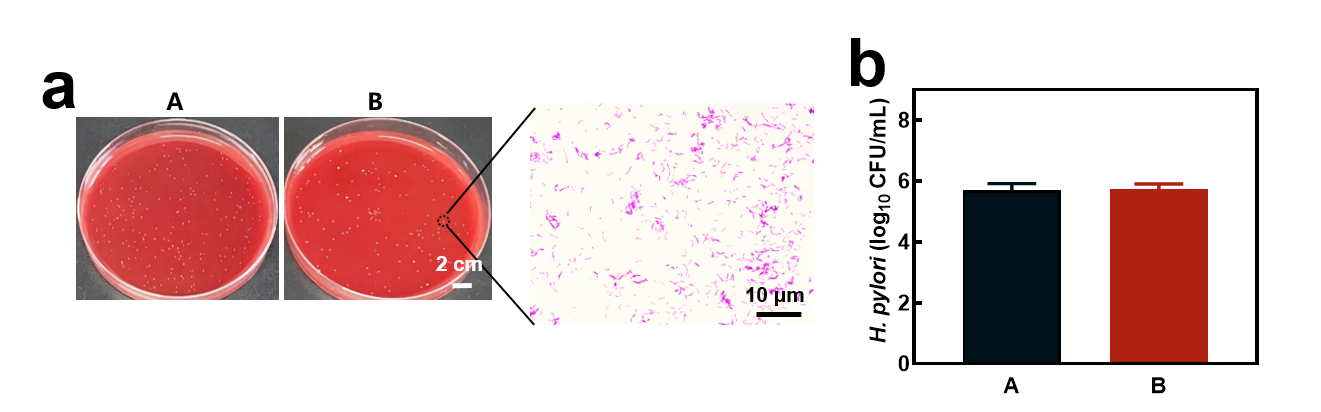


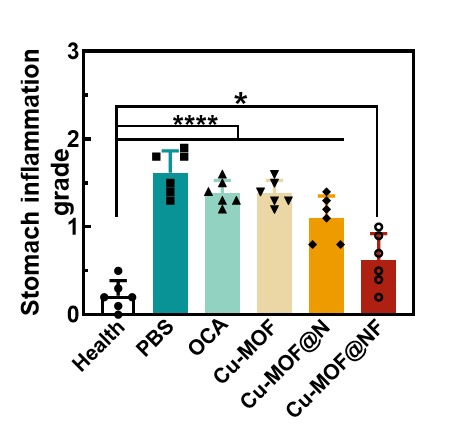
**Figure S20.** a) Plate plots and Gram staining plots of *H. pylori* colonies in the gastric tissues of mice. b) Quantification of *H. pylori* bacterial activity in a). Data are means ± s.d. (n ≥ 3).

**Figure S21.**The gastric tissue pathology scoring. Data are means ± s.d. (n = 6). **p* < 0.05, *****p* < 0.0001.

**
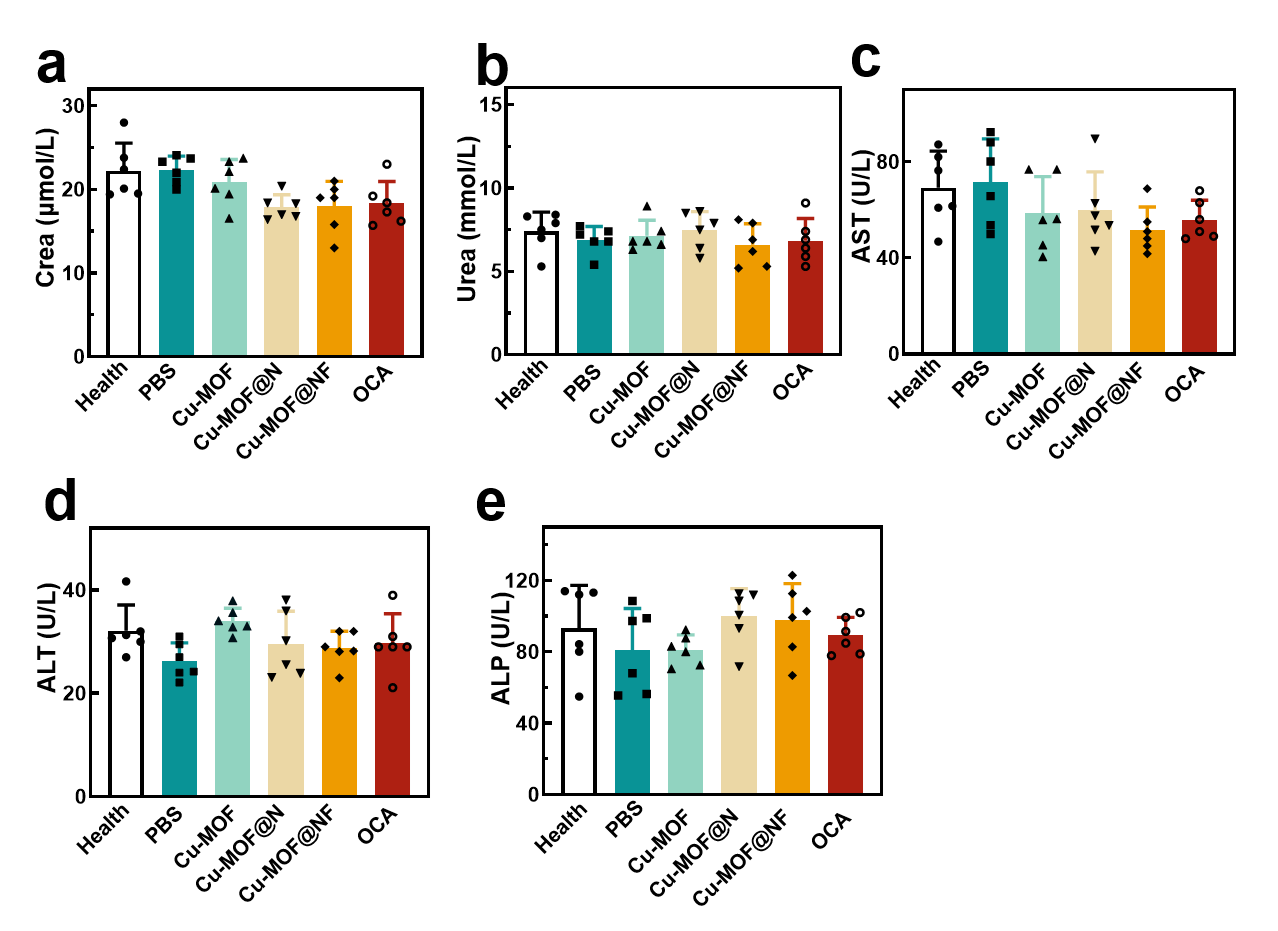
**

**Figure S22.** Plasma levels of creatinine (a), urea (b), aspartate aminotransferase (c), alanine aminotransferase (d), and alkaline phosphatase (e) in mice after treatment. Data are means ± s.d. (n = 6).


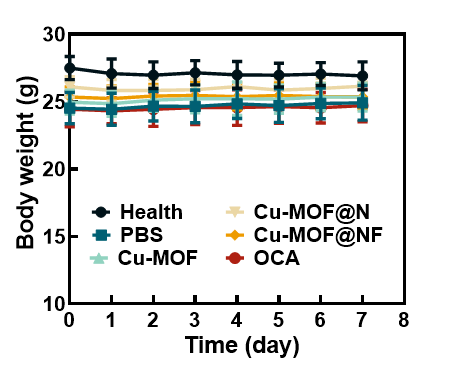


**Figure S23.** Body weight changes of mice during treatment. Data are means ± s.d. (n = 6).


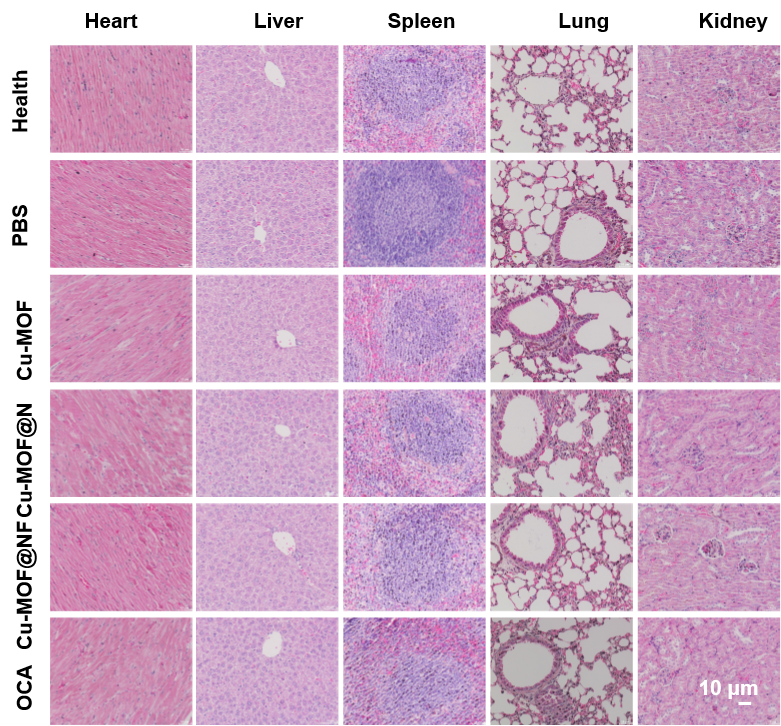


**Figure S24.** H&E staining of the major organs of mice.


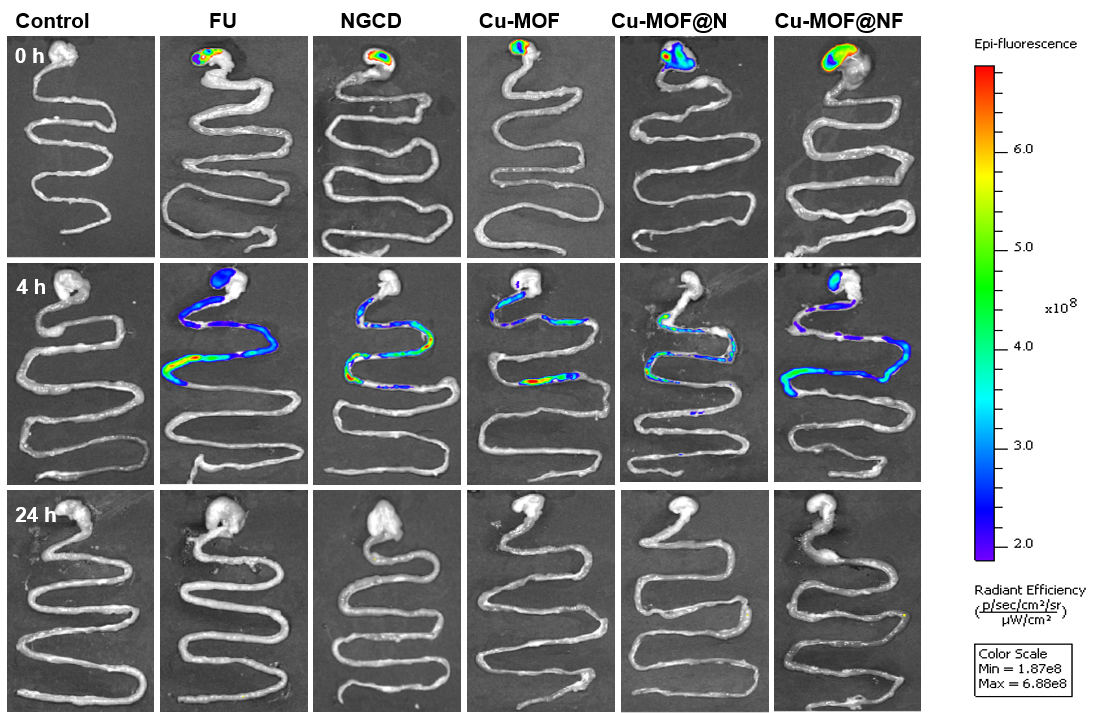


**Figure S25.** *H. pylori*-infected mice were given FITC-labeled nanomaterials by gavage, and the mice were executed after 0, 4, and 24 hours, respectively. Gastrointestinal tissues were imaged by the IVIS imaging system. Data are means ± s.d. (n ≥ 3).


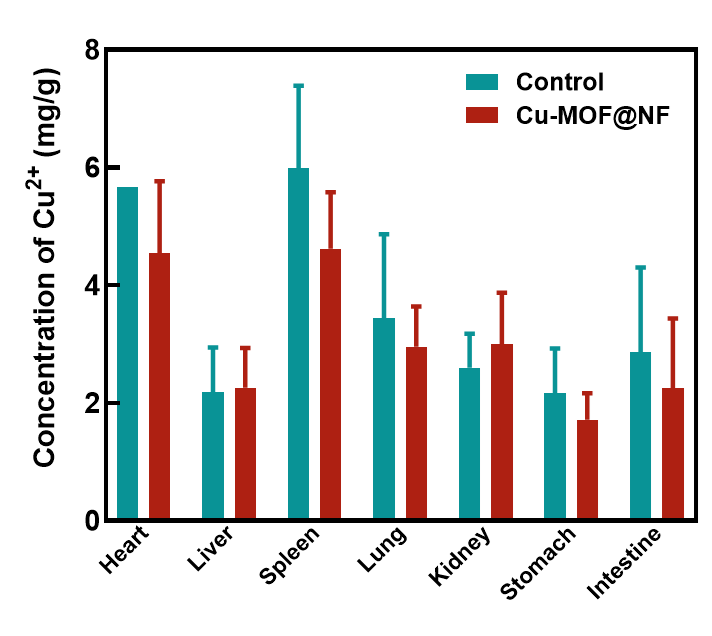


**Figure S26.** Concentration of Cu^2+^ in major organs (heart, liver, spleen, lung, kidney, stomach, and intestine). Data are means ± s.d. (n = 3).

**Table S1.** The ICP-AES results of Cu-MOF@NF.

| Sample name | Test items | Test results (mg/L) |
| --- | --- | --- |
| Cu-MOF@NF (200 mg/L) | Cu | 11 |

**Table S2.** Primers used for RT-qPCR.

| Primer name | Primer sequence Forward | Primer sequence Reverse |
| --- | --- | --- |
| GAPDH | CCTCGTCCCGTAGACAAAATG | TGAGGTCAATGAAGGGGTCGT |
| IL-1β | ATGATGGCTTATTACAGTGGCAA | GTCGGAGATTCGTAGCTGGA |
| IL-8 | ACTGAGAGTGATTGAGAGTGGAC | AACCCTCTGCACCCAGTTTTC |
| TNF-α | GAGGCCAAGCCCTGGTATG | CGGGCCGATTGATCTCAGC |
| IL-6 | AAGTCCGGAGAGGAGACTTC | TGGATGGTCTTGGTCCTTAG |
